# De novo human brain enhancers created by single-nucleotide mutations

**DOI:** 10.1126/sciadv.add2911

**Published:** 2023-02-15

**Authors:** Shan Li, Sridhar Hannenhalli, Ivan Ovcharenko

**Affiliations:** ^1^ Computational Biology Branch, National Center for Biotechnology Information, National Library of Medicine, National Institutes of Health, Bethesda, MD 20892, USA.; ^2^ Cancer Data Science Laboratory, Center for Cancer Research, National Cancer Institute, National Institutes of Health, Bethesda, MD 20892, USA.

## Abstract

Advanced human cognition is attributed to increased neocortex size and complexity, but the underlying evolutionary and regulatory mechanisms are largely unknown. Using human and macaque embryonic neocortical H3K27ac data coupled with a deep learning model of enhancers, we identified ~4000 enhancer gains in humans, which, per our model, can often be attributed to single-nucleotide essential mutations. Our analyses suggest that functional gains in embryonic brain development are associated with de novo enhancers whose putative target genes exhibit increased expression in progenitor cells and interneurons and partake in critical neural developmental processes. Essential mutations alter enhancer activity through altered binding of key transcription factors (TFs) of embryonic neocortex, including ISL1, POU3F2, PITX1/2, and several SOX TFs, and are associated with central nervous system disorders. Overall, our results suggest that essential mutations lead to gain of embryonic neocortex enhancers, which orchestrate expression of genes involved in critical developmental processes associated with human cognition.

## Teaser

Single nucleotide mutations led to the acquisition of neocortex enhancers and evolution of human cognition.

## INTRODUCTION

The neocortex is a mammalian innovation enabling complex cognitive and motor tasks (*1*, *2*). The substantial expansion and functional elaboration of the neocortex provides an essential basis for the advanced cognitive abilities of humans (*1*), which includes an increase in the proliferative capacity of the progenitor cells (*3*–*5*), an increase in the duration of their proliferative, neurogenic, and gliogenic phases (*6*, *7*), an increase in the number and diversity of progenitors, modification of neuronal migration, and establishment of new connections among functional areas (*1*).

Critical events in corticogenesis, including specification of cortical areas and differentiation of cortical layers, require precise spatiotemporal orchestration of gene expression (*8*). Modifications in gene regulation are thus hypothesized to be a major source of evolutionary innovation during cortical development (*1*, *8*, *9*). Among these are gain and loss of enhancers, repurposing of existing enhancers, rewiring of enhancer-gene interaction networks, and modifications of cross-talk between enhancers operating within the same cis-regulatory landscape (*10*). However, several fundamental questions remain open: to what extent the evolutionary gain and loss of enhancers has contributed to human-specific features of corticogenesis? Specifically, how often enhancer gain is associated with an increased expression of the target gene involved in human corticogenesis? Several studies have shown that single-nucleotide mutations could underlie enhancer gain/loss through disruption/creation of transcription factor (TF) binding sites during evolution (*11*, *12*). Therefore, to what extent the emergence of human-specific enhancers could be explained by a single or a few single-nucleotide mutations? How often do these mutations establish an enhancer from neutral DNA through creation of binding sites of activators as opposed to the disruption of binding sites of repressors? What are the TFs mediating critical enhancer gains and losses and what gene regulatory networks are induced by these mutations? A previous study identified Human Gained Enhancers (termed HGEs) (*13*) that exhibit increased regulatory activity in human relative to macaque and mouse. In contrast, our focus is de novo gained enhancers in human that presumably originate from neutral noncoding sequence via minimum number of single-nucleotide substitutions along the human lineage. Besides the availability of enhancer activity profiles in the developing brain of humans and macaques (*13*), a quantitative model that can accurately estimate enhancer activity from DNA sequence, with single-nucleotide sensitivity, is critical to answering the questions above.

In this study, we developed a deep learning model (DLM) able to learn the sequence encryption of primate embryonic neocortex enhancers, enabling us to quantify the functional effect of single-nucleotide mutations on enhancer activity. Leveraging the recently available enhancer activity profiles in developing neocortex in humans and macaques (*13*) and the DLM-predicted enhancer activities in both organisms and their predicted common ancestor (*14*), we identified single-nucleotide mutations that potentially drive human-specific regulatory innovations. Our model-based analysis suggests that a single-nucleotide mutation might be sufficient to give rise to an enhancer, leading to increased expression of the proximal target gene. As a group, de novo gained enhancers induce genes that are critical to cognitive function and are expressed preferentially in the progenitor and interneuron cells of the developing neocortex. De novo gained enhancers and their target genes induce and mediate a potential core regulatory network in the developing human neocortex, with POU3F2 occupying a central position. Essential single-nucleotide mutations that are predicted to be resulting in de novo enhancer gain exhibit relaxed negative, or potentially adaptive, selection. The essential mutations and de novo gained enhancers are enriched for cognitive traits; in particular, the de novo gained enhancers associated with regulation of key TFs are enriched for de novo mutations in patients with the autism spectrum disorder (ASD). Compared to HGEs, although de novo gained enhancers have relatively weaker enhancer activity, they are more likely to be functional in the developmental human brain based on experimentally validated brain enhancers (*15*). In addition, the de novo enhancers are more likely to turn on gene expression in human and regulate genes associated with brain development. Integrating a DLM with epigenomic data allowed us not only to identify de novo gained human-specific enhancers that might underlie advanced cognition but also to gauge the impact of single-nucleotide mutations in this process.

Overall, our results, on the basis of the H3K27ac profiles in developing human and macaque brain, and a sequence-specific DLM of embryonic neocortical enhancers, suggest a widespread de novo gain in enhancers, which could largely have been created by a single-nucleotide mutation according to our model, in the progenitors and interneurons of the developing human neocortex, that together induce a core regulatory network that associated with human cognitive abilities and cognitive disorders.

## RESULTS

### Our pipeline identifies de novo enhancer gains and the underlying essential human mutations

**Fig. 1.**
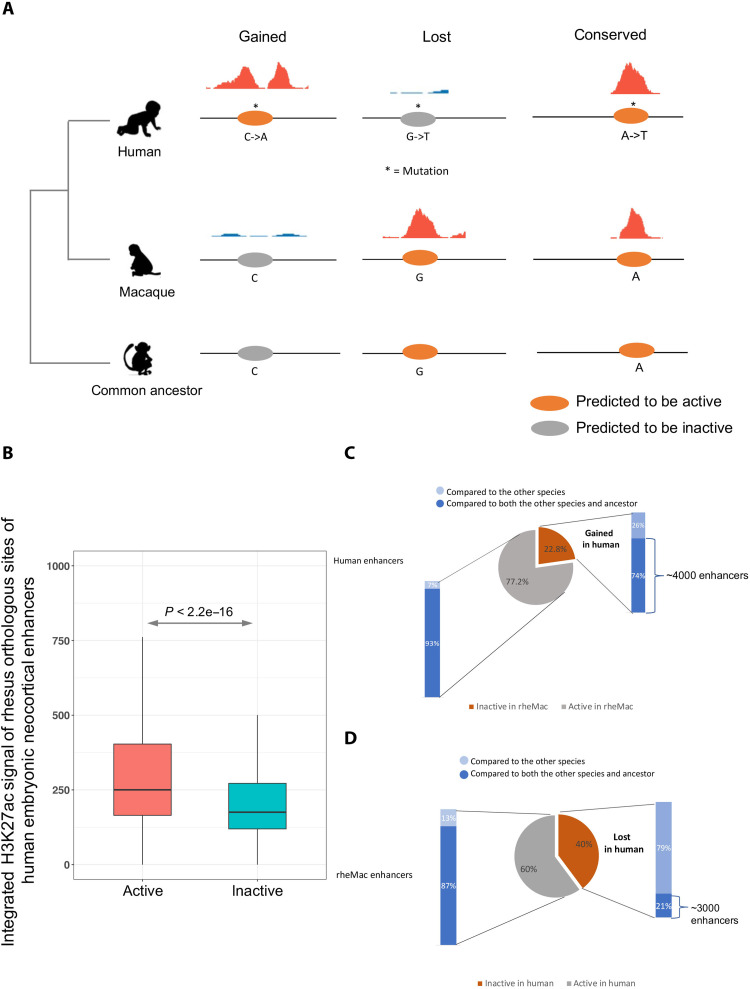
De novo gained and lost enhancers. (**A**) Identification of de novo gained, lost, and conserved enhancers. If a human enhancer scored highly by the DLM and scored low both in macaque and in the common ancestor, and was not detected by H3K27ac in macaque, it was considered to be gained in humans. If a macaque enhancer with a high DLM score scored high in common ancestor, scored low in human, and was undetectable by H3K27ac in human, it was considered a loss in human. The enhancers that are detected by H3K27ac in both human and macaque and scored highly in all three genomes were called conserved enhancers. (**B**) Comparison of embryonic macaque neocortex integrated H3K27ac signal intensities (within the 1-kb enhancers) between the predicted active and inactive macaque orthologs of human embryonic neocortex enhancers. (**C**) Fraction of de novo gained human embryonic neocortex enhancers by comparing human to both macaque and their common ancestor. Specifically, 74% of human enhancers that are inactive in macaque are active in the common ancestor and 93% of human enhancers that are active in macaque are active in the common ancestor. (**D**) Fraction of lost human embryonic neocortex enhancers by comparing human to both rhesus macaque and their common ancestor. Specifically, 21% of macaque enhancers that are inactive in human are active in the common ancestor and 87% of macaque enhancers that are active in human are active in the common ancestor. Light blue refers to relative to the other species, and dark blue refers to relative to both the other species and common ancestor.

To assess functional impact of single-nucleotide mutations on enhancer activity, we leveraged the H3K27ac chromatin immunoprecipitation sequencing (ChIP-seq) data profiling human and macaque corticogenesis as a proxy for active enhancers (*13*) and built a DLM to learn the regulatory code encrypted in the enhancer sequences (fig. S1, A to C, and Methods). Next, to identify human-specific de novo gain and loss of enhancers, in addition to using the observed enhancer activities in human and macaque, we also integrated the DLM-predicted enhancer activities in human, macaque, and the human-macaque common ancestor inferred from multiple sequence alignment (*14*); incorporation of predicted enhancer activity in the common ancestor enabled us to differentiate gains in human from losses in macaque (Fig. 1A and Methods). We then prioritized the single-nucleotide human-macaque mutations in the de novo gained and lost enhancers based on the difference of the DLM scores between the macaque sequence and the intermediate sequence with one or more introduced human allele(s). For an enhancer with multiple mutations, which was either gained or lost in the human genome, we first introduced each human-specific allele to its matching macaque sequence and estimated its impact on enhancer activity using the difference in the DLM score attributed to the human allele. By iteratively increasing the number of introduced human-specific alleles and scoring the modified sequence, we evaluated the impact of combinations of mutations and determined the minimal number of mutations needed for an enhancer to be gained or lost in the human lineage.

### The DLM can accurately predict embryonic neocortex enhancers in human and macaque

The human embryonic neocortex H3K27ac ChIP-seq peaks were obtained from the four temporal/spatial groups: the whole cortex at 7 post-conception weeks (p.c.w.; CS16) and 8.5 p.c.w. (CS23) and primitive frontal and occipital tissues from 12 p.c.w. (F2F and F2O) (*13*). We trained a DLM separately for each set of enhancers (Methods). The DLM was able to discriminate human embryonic neocortex enhancers from accessible regions devoid of non–fetal brain enhancer with high accuracy: the area under the receiver operating characteristic curve (auROC) ranges from 0.9 to 0.94 (fig. S1B), and the area under the precision-recall curve (auPRC; expectation = 0.091) ranges from 0.56 to 0.63 for the four datasets (fig. S1C). The consistently high accuracy of all models showed the ability of DLMs in capturing sequence signatures of brain enhancers similarly to previous modeling of enhancers in other cells and tissues (Supplementary Results 1), and prompted us to conjecture that the four groups of enhancers tend to share either genomic locations or sequence characteristics. To assess their sequence similarity, we trained the DLM on one set and predicted those from all other sets. We observed both high auROCs and auPRCs (fig. S1D), strongly suggesting shared sequence characteristics across the four enhancer sets. However, the genomic overlap between any two groups of enhancers is relatively low (20 to 40%; fig. S1E), indicating that the four sets of enhancers overlap only partially but share sequence characteristics.

We proceeded to investigate the de novo gain and loss of enhancers by comparing human 8.5 p.c.w. (CS23) sample and macaque sample at approximately matching time point (e55) (*13*), as the DLM trained on CS23 has not only high auROC (0.92) but also the highest precision at a low false-positive rate (FPR = 0.1; fig. S1, B and C). For the 4066 de novo gained enhancers (CS23), we found that 828 (20.4%) are active at the CS16 (p.c.w. 7) time point, 1816 (44.7%) are active at F2F (p.c.w. 12 time point, frontal lobe), and 1550 (38.1%) are active at F2O (p.c.w. 12 time point, occipital). For the 2925 lost enhancers (CS23), 2583 (88.3%) are inactive at CS16, 2237 (76.5%) are inactive at F2F, and 2246 (72.8%) are inactive at F2O. Therefore, de novo gained and lost enhancers are both dynamic to varying degrees. In particular, the de novo gained enhancers are more time point specific. To ascertain that the DLM trained on CS23 can accurately predict the enhancer activity in macaque, we scored the macaque orthologs of CS23 enhancers and compared the e55 H3K27ac signal intensities of the macaque orthologs predicted to be active with those predicted to be inactive (Methods). The predicted active regions have significantly stronger H3K27ac signal (Fig. 1B), suggesting that the DLM learned from human embryonic neocortical enhancers can accurately gauge the enhancer activity in macaque from its genomic sequence.

We next identified the enhancers de novo gained, lost, or conserved in human relative to both macaque and human-macaque common ancestor based on the H3K27ac profile and DLM scores (Methods and Fig. 1A). In total, we identified 4066 de novo gained (Fig. 1C), 2925 lost, and 23,119 conserved neocortical enhancers (Fig. 1D). Although most of the developmental neocortical enhancers remained active since the divergence of human and macaque from their common ancestor, there are certain groups of enhancers that are gained or lost in the human lineage, prompting us to conjecture that these gain and loss events may correlate with the human-specific features of corticogenesis, which we investigate next.

### De novo gained enhancers are associated with critical cortical developmental functions

**Fig. 2.**
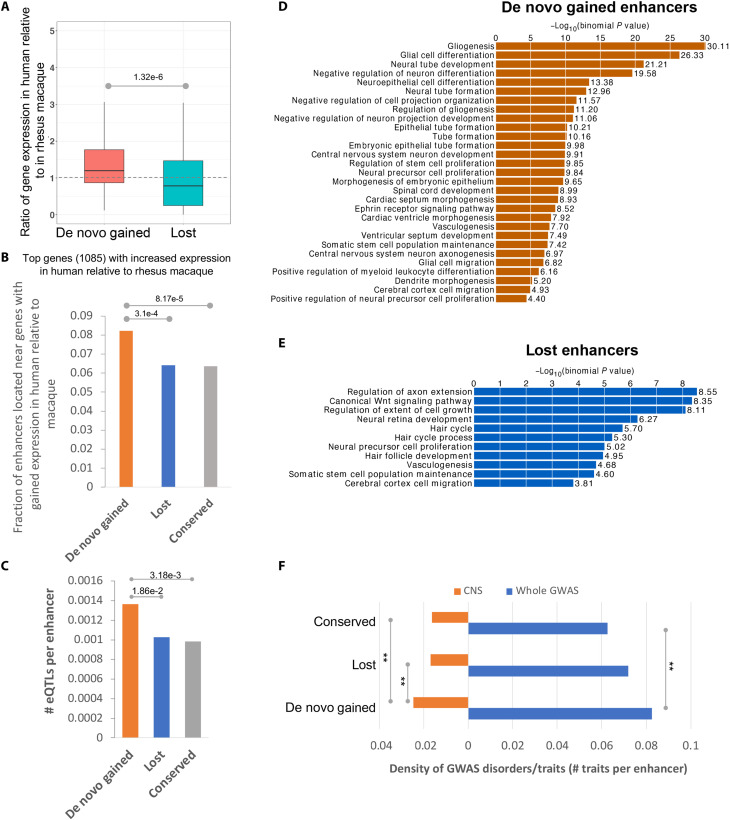
De novo gained enhancers are associated with essential biological pathways. (**A**) The expression level of genes near the de novo gained enhancers is increased. (**B**) Gained enhancers are enriched near the genes that are mostly highly expressed in humans as compared to rhesus macaque. (**C**) Average number of eQTLs per enhancer. (**D**) Biological processes that are associated with gained enhancers based on whole-genome region enrichment analysis performed using the GREAT tool (*57*). (**E**) Biological processes that are associated with lost enhancers based on GREAT whole-genome region enrichment. (**F**) The CNS-related GWAS traits are enriched in the gained enhancers compared to both lost and conserved enhancers.

Next, to investigate whether de novo enhancer gains are accompanied by an increase in the expression of their putative target genes, we compared the human-to-macaque ratios of gene expression near gained enhancers versus those near lost enhancers and observed that the genes near gained enhancers show a human-specific increase in expression, while a reverse trend is exhibited by genes near lost enhancers (Fig. 2A); this trend holds when we rely on Hi-C contact data to map an enhancer to its target genes (fig. S2). Consistently, gained enhancers are enriched near the genes with top 5% highest expression relative to macaque (Fig. 2B). Notably, the fetal brain expression quantitative trait loci (eQTLs) (*16*) are significantly enriched in de novo gained enhancers compared to lost and conserved enhancers (Fig. 2C and fig. S3), suggesting that these de novo loci are more likely to have regulatory functions. These results together support a causal link between enhancer gain and an increase in the expression of their target genes. Furthermore, the de novo gained enhancers are primarily associated with gliogenesis, neural tube development, and neural precursor cell proliferation, among other central nervous system (CNS)–related developmental processes (Fig. 2D, fig. S4A, and table S1). In contrast, lost enhancers are associated with only a small number of CNS-related essential biological processes, including regulation of axon extension, neural retina development, neural precursor cell proliferation, and cerebral cortex cell migration (Fig. 2E, fig. S4B, and table S2). Lost enhancers are enriched for far fewer processes than the de novo gained enhancers (Fig. 2, D and E); at a stringent enrichment *P* value threshold of 10^−9^, lost enhancers are not enriched for any process, while gained enhancers are enriched for 17 functions (Fig. 2, D and E). As expected, conserved enhancers, which constitute most (72%) of all enhancers considered, are enriched for a large range of CNS developmental processes (fig. S5A). Last, we found that CNS-related Genome-Wide Association Studies (GWAS) traits (tables S3 to S5) are enriched among de novo gained enhancers compared to conserved and lost enhancers (Fig. 2F), suggesting an essential role of de novo gained enhancers in establishing cognitive traits.

**Fig. 3.**
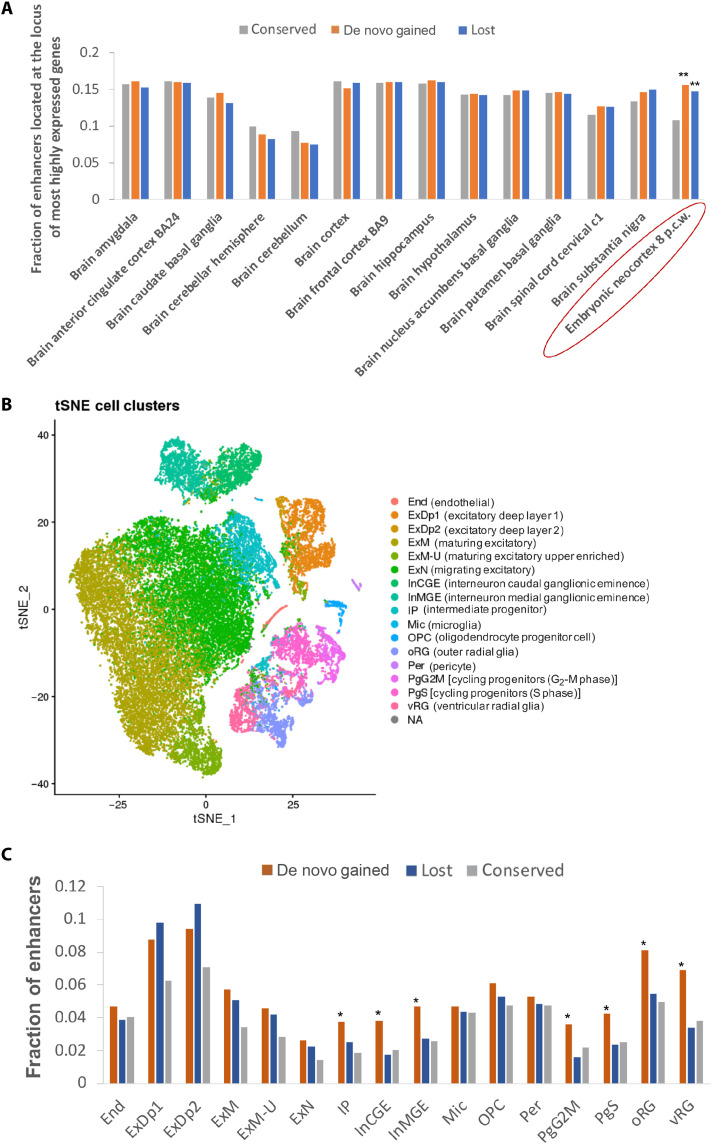
The de novo gained enhancers are enriched in the progenitor cells and interneurons. (**A**) The de novo gained enhancers are significantly enriched in the most highly expressed genes of embryonic human neocortex but no other adult brain regions. **P < 1 × 10^−3^, Fisher’s exact test. (**B**) Scatterplot visualization of cells after principal components analysis and t-distributed stochastic neighbor embedding (tSNE), colored by Seurat clustering and annotated by major cell types. (**C**) Fraction of enhancers near genes that are most highly expressed in all the cell clusters.

We further observed that, relative to conserved enhancers, de novo gained and lost enhancers are significantly enriched near genes that are specifically expressed in the embryonic neocortex (8 p.c.w.), but not adult brain (Fig. 3A and Methods), implicating them specifically in brain development. To fine map gained and lost enhancer activities to specific cell types of the developing human brain, we leveraged the single-cell transcriptomic data of developing human neocortex during midgestation (*17*), as the transcriptome at early gestation (8 p.c.w.) and midgestation (17 p.c.w.) stages is tightly correlated [Pearson Correlation Coefficient (PCC) = 0.934, Spearman correlation = 0.925]. Among the 16 transcriptionally distinct cell types/states (Fig. 3B), de novo gained enhancers are primarily enriched near the genes specifically expressed in progenitor cells including radial glia (oRG, vRG), cycling progenitors in G_2_-M phase (PgG2M) and S phase (PgS), intermediate progenitors (IP), and interneurons (InCGE and InMGE), which connect different brain regions and are involved in cell/axon migration (Fig. 3C and fig. S6). Although lost enhancers are enriched near genes specifically expressed in excitatory neurons (excitatory deep layers ExDp1 and ExDp2, maturing excitatory neurons ExM, ExM-u, and migrating excitatory neurons ExN), de novo gained enhancers also exhibited a comparable level of enrichment in the same loci, thus arguing for compensatory impact on either the target gene expression or the phenotypic change to a large extent. Thus, the unique enrichment of de novo gained enhancers in the progenitor cells and interneurons might have contributed to the expansion of cortical surface and to an increased complexity of connections in the human cerebral neocortex, both of which together underpin the advanced cognition in humans. Hence, in the following, we focus specifically on the de novo gained enhancers and investigate their emergence and functional consequences.

### De novo enhancer gain may often be attributable to a single essential mutation

**Fig. 4.**
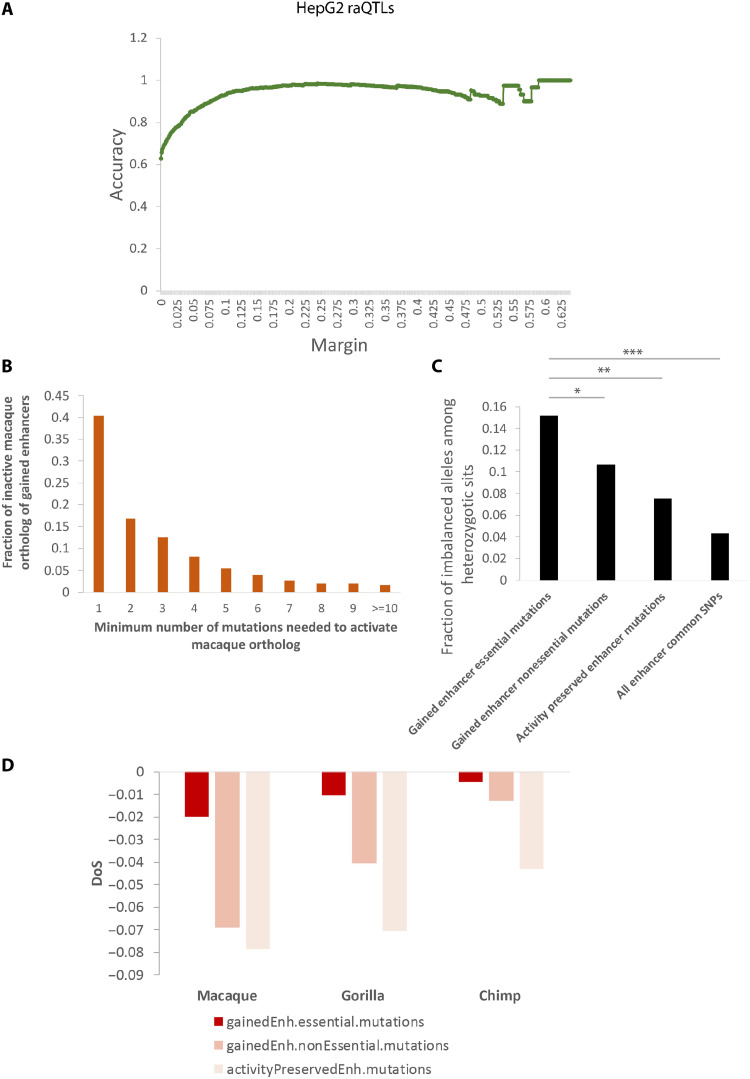
Essential mutations show larger impact on enhancer activity. (**A**) Deep learning enhancer classifiers in HepG2 accurately predicted allele-specific effects on enhancer activity (the allele with stronger enhancer activities). The predictions were evaluated with regulatory activity QTLs (raQTLs) identified in HepG2 cell lines (*52*). Margin shown on the *x* axis is the threshold of predicted probability differences between the two alleles for classifying high-confidence predictions. Performance is measured by accuracy (*y* axis) of predicting the allele with higher enhancer activities based on DLM score difference above certain threshold (*x* axis). (**B**) Fraction of de novo gained enhancers that could be activated by specific number of mutations. (**C**) Fraction of mutation/SNP sites that are in allelic imbalance. *P ≤ 0.05, **P ≤ 0.01, ***P ≤ 1 × 10^−3^, Fisher’s exact test. (**D**) DoS score of the mutated sites, using macaque, gorilla, and chimp as comparison species.

To investigate the extent to which the enhancer gains could be explained by single-nucleotide mutations and to identify the minimal number of mutations needed to activate a neutral DNA sequence, we first compared the number of human-macaque mutations in de novo gained and conserved enhancers. The number of human-macaque mutations in de novo gained and conserved enhancers is comparable—~50 in a 1-kb enhancer (fig. S7). Recall that our DLM is trained to distinguish fetal brain enhancers from accessible non–fetal brain enhancer regions and not necessarily to assess the effect of single-nucleotide changes. Therefore, we first performed a series of analyses to ensure that the DLM score (i) tracks enhancer activity and (ii) can accurately predict allele-specific effects on enhancer activity (Fig. 4A and Supplementary Results 2). To identify critical mutations, we applied our DLM to prioritize human-macaque mutations in de novo gained enhancers based on the mutations’ impact on enhancer activity by iteratively introducing them into the potentially inactive macaque sequence orthologous to human CS23 enhancers. We were thus able to assess the minimal number of mutations capable of activating an enhancer (Methods). Although only ~1.8% of all mutations in de novo gained enhancers are independently able to activate an enhancer (we call these essential mutations), ~40% of the de novo gained enhancers contain at least one essential mutation (Fig. 4B). As expected, the smaller the minimal number of mutations needed to create an enhancer, the larger is their individual impact as per the DLM (fig. S8). To validate the impact of essential mutations on enhancer activity, we assessed their allelic imbalance of H3K27ac reads at the heterozygous sites. We hypothesized that the human reference allele at essential positions should exhibit larger H3K27ac read coverage than the macaque reference allele (Methods). Compared to three other groups of mutations/single-nucleotide proteins (SNPs) as controls, essential mutation positions are significantly associated with imbalance of H3K72ac reads coverage with the human reference allele (Fig. 4C). Consistent results were observed on the basis of the deoxyribonuclease (DNase) I–hypersensitive site (DHS) profile at similar developmental time points (fig. S9) (*18*, *19*). Furthermore, compared to nonessential mutations and common SNPs, the essential mutations are more enriched near tissue-specific genes (fig. S10A and table S6) and are more likely to be associated with gain of gene expression in human as compared to macaque (fig. S10B). These results strongly support a causal link between the essential mutations and enhancer gain.

We next examined the evolutionary constraints on essential mutations by applying the direction of selection (DoS) (*20*) test, which is a refinement of McDonald-Kreitman test (*20*), to measure the direction and degree of departure from neutral selection (Methods). DoS test is applied to a pair of species, and a positive and negative DoS indicate positive and negative selection, respectively. We estimated the DoS values for three sets of mutations—essential mutations, nonessential mutations in de novo gained enhancers, and mutations within activity preserved enhancers (Methods)—comparing human with macaque, gorilla, and chimp. As shown in Fig. 4D, compared to other mutation classes, essential mutations have the highest DoS values, accordant with a relaxed negative selection, or potentially a subset of sites being under positive selection, both of which manifest as accelerated evolutionary rate (*21*–*25*). Although only a very small minority of human-macaque mutations (<2.5%) are polymorphic, among the small fraction of essential mutations that are polymorphic, we found that their derived allele frequencies are significantly higher than those for controls (fig. S11A), which is consistent with a relaxed purifying selection and together corroborate the accelerated evolutionary rate of the essential mutation sites.

### Essential mutations are associated with cognition and neurodevelopmental disorders

**Fig. 5.**
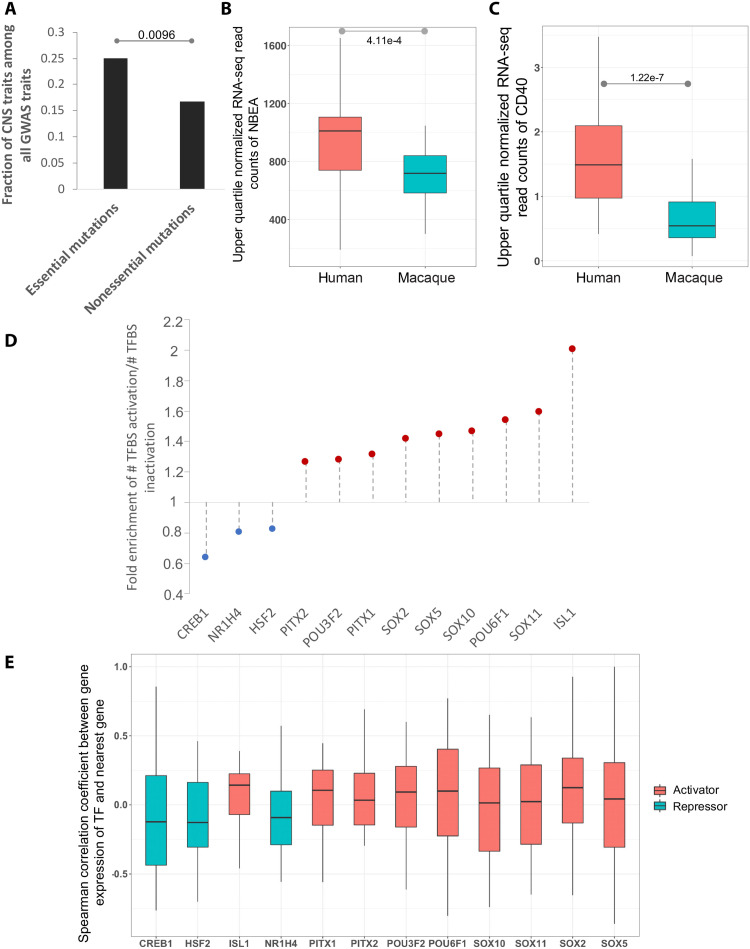
Essential mutations are associated with cognition-related traits and tend to create binding sites of activators. (**A**) Fraction of GWAS traits at the mutation sites that are CNS-related. (**B**) Comparison of trimmed mean of M values (TMM)-normalized expression of *NBEA* between embryonic human and rhesus macaque individuals. *P* values are based on the Wilcoxon test. (**C**) Comparison of TMM-normalized expression of *CD40* between embryonic human and rhesus macaque individuals. *P* values are based on the Wilcoxon test. (**D**) Enrichment of ratio of binding site gain to loss caused by essential mutations overlapping enriched TFBSs as compared to those caused by common SNPs. (**E**) Spearman correlation coefficient of expression between the cognate TF of essential mutation and its nearest gene.

Given our observation that the essential mutations are causally linked to enhancer activity in the embryonic neocortex, we assessed whether the essential mutations are preferentially associated with CNS-related GWAS traits (Methods). We observed a ~1.5-fold enrichment of CNS-related traits at the essential mutation positions as compared to nonessential mutation sites (Fig. 5A and tables S7 and S8). Specifically, 7 of 28 GWAS traits overlapping essential mutations are CNS-related, and, more importantly, 6 of those are associated with cognition (table S7). We further investigated three such cases where the nearest genes are protein-coding genes with available expression data at approximate developmental stages (*26*).

One essential mutation site coinciding with the common SNP *rs9574096* is tightly linked to the tag SNP (*rs9574095*; correlation = 0.93) associated with the trait “Mathematical ability.” Both variants are located in the intronic region of the gene *neurobeachin* (*NBEA*), which is an autism-linked gene that fine-tunes signals at neuronal junctions (*27*). Mice missing one copy of *NBEA* show autism-like behavior (*27*). We found that *NBEA* exhibits a significantly higher embryonic neocortex expression in human compared to macaque at a similar early developmental stage (Fig. 5B) (*26*). The macaque allele *A* appears to be bound by another autism risk TF, RFX3 (*28*), whereas the human allele *T* does not (Methods), suggesting a loss of RFX3 binding resulting in an increased enhancer activity and *NBEA* gene expression. Consistently, *RFX3* expression is negatively correlated with that of *NBEA* in the embryonic neocortex across human and macaque individuals (Spearman rho = −0.26). In addition, *NBEA* is specifically expressed in sub-brain regions including excitatory neurons (ExDp1, ExDp2, ExM, and ExM-U) and interneurons (InMGE) (*17*), suggesting a link between these sub-brain regions and autism.

Other two essential mutation positions coincide with two common SNPs *rs747759* and *rs1535043*, both of which are in perfect linkage disequilibrium (LD) with each other. Notably, *rs747759* is the tag SNP of the GWAS trait “Neuroticism.” The nearest gene of the two SNPs is *CD40*, which again displays a much higher expression in humans as compared to macaque (Fig. 5C). CD40 is a major regulator of dendrite growth and elaboration in the developing brain (*29*) and contributes to synaptic degeneration in Alzheimer’s disease (AD) (*30*), which may have developmental origins (*31*). The human allele *T* at the tag SNP *rs747759* causes either a potential binding site gain of NFYA or a potential binding site loss of NHLH1 (table S9). *NFYA* is an AD-associated gene (*32*–*34*). On the other hand, NHLH1 is known to play important roles in neuronal and glial differentiation and maturation (*35*). However, the chance for NHLH1 to be a repressor of *CD40* is dampened by their strong positive correlation of gene expression across human and macaque individuals (Spearman rho = 0.58). By contrast, *NFYA* expression is positively correlated with *CD40* expression (Spearman rho = 0.29). At *rs1535043*, the human allele *T* is associated with the gain of an *EHF* binding site. However, its links with CNS traits are unclear. Together, these results suggest a link between essential mutations in de novo gained enhancers and cognition-related traits as well as neurodevelopmental disorders in humans.

### Essential mutations tend to create binding sites of key activating TFs of developing human brain

Next, we investigated the relative prevalence and importance of binding site gain versus loss in the de novo gained enhancers. Toward this, we focused on the TFs whose binding sites are enriched in the de novo gained enhancers compared to the conserved ones (using both human and macaque sequences to avoid allelic bias; table S10) and quantified the global tendency of essential mutations to lead to binding site gain versus loss (Methods). Overall, we observed that nine TFs including POU3F2, PITX2, PITX1, SOX2, SOX5, SOX10, POU6F1, SOX11, and ISL1 tend to gain binding sites mediated by essential mutations in human (Fig. 5D), suggesting an activator role of these TFs. Conversely, three TFs, CREB1, HSF2, and NR1H4, are more likely to lose their binding sites (Fig. 5D), suggesting potentially repressive roles. Moreover, the overall positive or negative correlation of gene expression between these putative cognate TFs of the essential mutations and their nearest genes further validates their activator or repressor roles, respectively (Fig. 5E). In short, the de novo gained enhancers are more likely to be activated by the creation of binding sites of activators due to the essential mutations.

### De novo gained enhancers induce a potential human-specific TF regulatory network

**Fig. 6.**
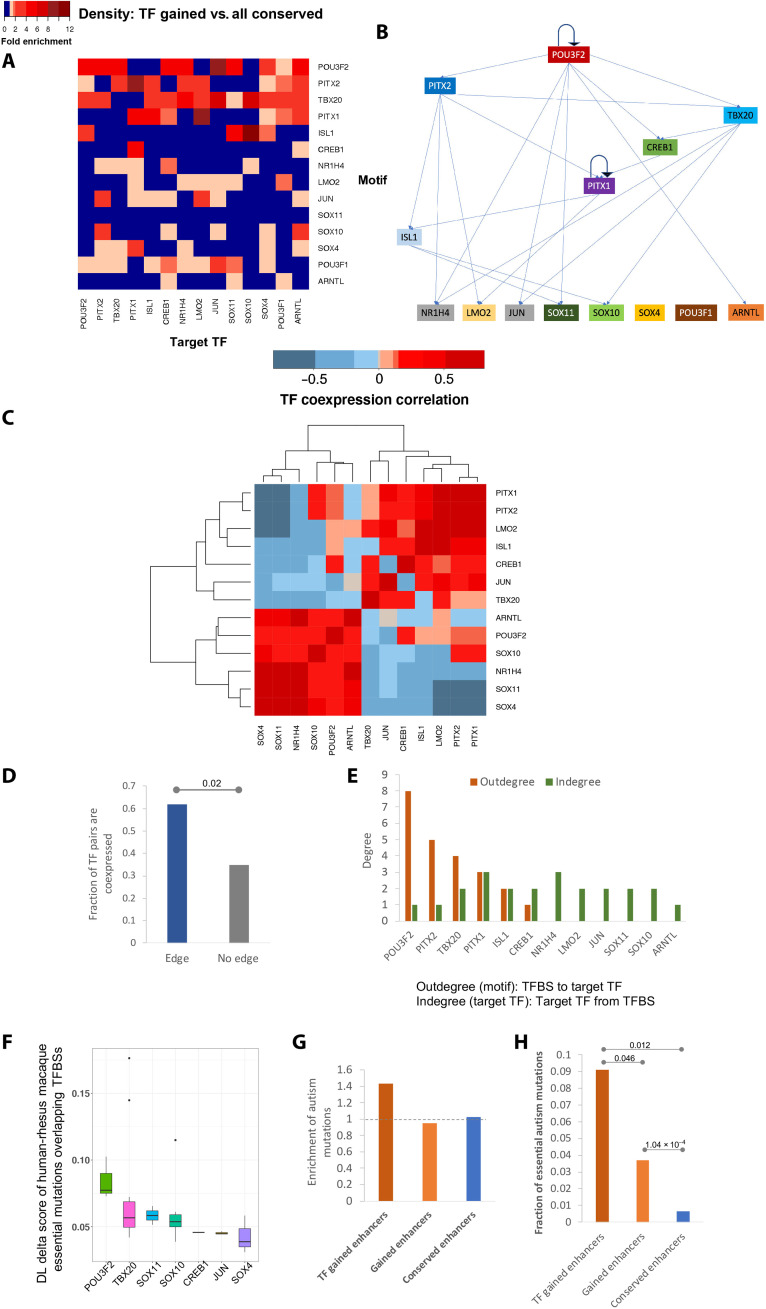
A hierarchical regulatory network of TFs induced by de novo gained enhancers. (**A**) Enrichment of density of TFBSs of the 14 TFs in the locus of the 14 TF genes. (**B**) Inferred hierarchical structure of the 14 TFs. (**C**) Spearman correlation coefficient of the 14 TF genes across the embryonic human and macaque individuals. (**D**) Comparison of fraction of TF pairs that are coexpressed (Spearman correlation coefficient > 0.3) between the pairs with links and those without links. *P* value is calculated using Fisher’s exact test. (**E**) Out-degree and in-degree of each TFs. (**F**) Distribution of DLM delta score caused by the essential mutations overlapping the 14 TFs. (**G**) Fraction of autism de novo mutations located within each set of enhancers normalized by the fraction of common SNPs falling into the same set of enhancers. (**H**) Fraction of autism de novo mutations within each set of enhancers, which are essential.

Transcriptional programs driving cell state are governed by a core set of TFs (also called master regulators), which auto- and cross-regulate each other to maintain a robust cell state. The ensemble of core TFs and their regulatory loops constitutes core transcriptional regulatory circuitry (*36*–*38*). The genes near de novo gained enhancers are enriched for transcriptional regulators (fig. S5B). We hypothesized that the TFs regulated by the de novo gained enhancers form a core regulatory network in the human embryonic neocortex. Toward this, first, we identified 24 TF genes (table S11) near de novo gained enhancers and performed a motif scan for each of the 14 TFs having a known binding motif among all enhancers near the 24 TF genes (Methods). We found that most of the 14 TF motifs are enriched in the de novo gained enhancers near TF genes compared to the conserved enhancers in the same loci (fig. S12), suggesting a core regulatory network formed by these TFs. Next, we established a putative regulatory relationship for each TF pair based on the enrichment of the density of one TF’s motif in the de novo gained enhancer near another TF, including autoregulation, using conserved enhancers associated with the 24 TFs as the background (Fig. 6, A and B). The inferred links are supported by our observation that linked TF pairs tend to have correlated expressions, as compared to those that are not (Fig. 6, C and D). On the basis of the number of TFs each TF regulates, POU3F2 is likely to be the master regulator, with PITX2, TBX20, and PITX1 playing critical roles (Fig. 6E). Moreover, we found the essential mutations that create a binding site for the TFs at higher hierarchical levels have a larger impact on the enhancer activity according to the DLM (Fig. 6F). The de novo noncoding mutations in autism patients (*39*) are specifically enriched in the set of de novo gained enhancers associated with TF activity (Fig. 6G). The de novo autism mutations within this subset of de novo gained enhancers are more likely to be essential, which alone can deactivate an enhancer, as compared to those other de novo gained and conserved enhancers (Fig. 6H). Together, these results suggest that essential mutations and the resulting enhancer gains may have helped create a core transcriptional regulatory network, with POU3F2 in a central position, to mediate a human-specific gene expression program in the developing human neocortex, associated with cognitive traits.

### De novo gained enhancers exhibit weaker enhancer activity but are more likely to be functional in the developing human brain compared to HGEs

Reilly *et al.* (*13*) defined HGEs based on a comparative analysis of enhancer-associated epigenetic marks (H3K27ac and H3K4me2) in human with rhesus macaque and mouse. HGEs are enhancers with increased activity in human as compared to both macaque and mouse. In sharp contrast, our “de novo” gained enhancers originate from presumably “neutral” noncoding sequence—they are detected in human but not in macaque and are predicted by the DLM to be inactive in both the macaque and the common ancestor of human and macaque (Methods). HGEs are largely a subset of what we consider conserved enhancers in our study (85% CS23 HGEs overlap our conserved enhancers) and not de novo gained enhancers (only 11.9% overlap de novo gained enhancers).

**Fig. 7.**
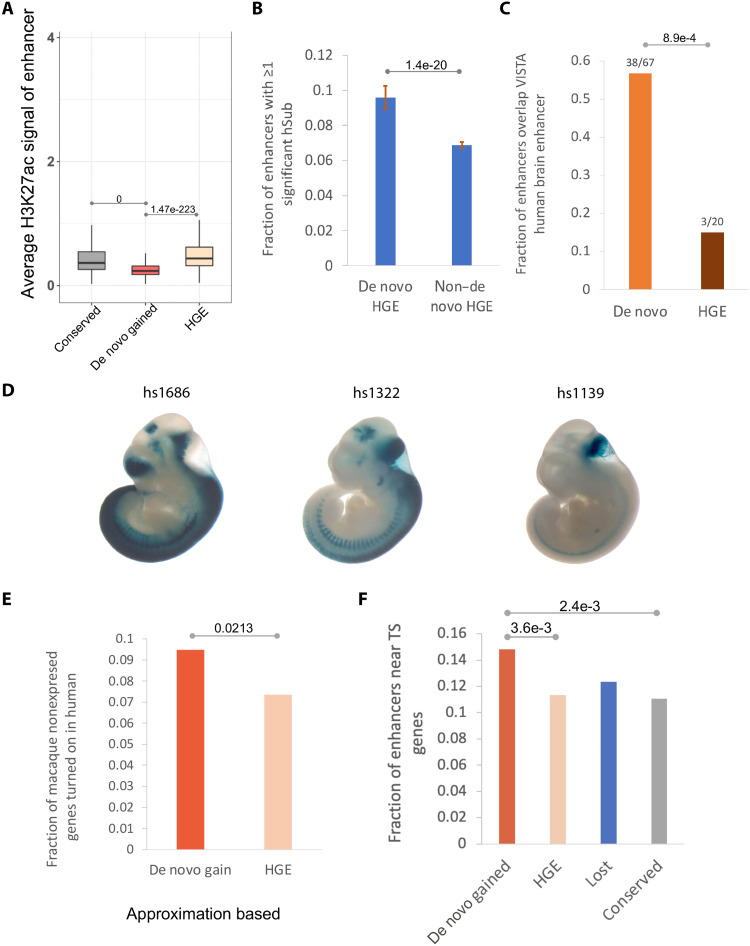
De novo gained enhancers versus HGEs. (**A**) De novo gained enhancers exhibit weaker enhancer signal. (**B**) Fraction of enhancers with ≥1 hSubs. Bar plot shows the median and SD of fraction of enhancers with at least one hSub by 90% bootstrapping for 50 times. *P* value is based on *t* test. De novo HGEs refer to HGEs that overlap de novo gained enhancers, and non–de novo HGEs refer to CS23 HGEs that do not overlap de novo gained enhancers. (**C**) For de novo gained enhancers and HGEs, the plots show among all enhancers that were experimentally tested in VISTA (denominator) the fraction that tested active in the developing brain (numerator). (**D**) Three examples of de novo enhancers overlapping validated VISTA human elements active in the developing brain. *hs1686*: active in forebrain, hindbrain, midbrain, and neural tube. *hs1322*: active in forebrain, midbrain, and hindbrain. *hs1139*: active in hindbrain. VISTA embryo staining images were retrieved from the VISTA Enhancer Browser at https://enhancer.lbl.gov/ (*15*). (**E**) Fraction of enhancers located near genes with very low expression [reads per kilobase of exon per million reads mapped (RPKM) < 1] in macaque and RPKM > 1 in human. (**F**) Fraction of enhancers that are near tissue-specific (TS) genes.

Notably, de novo gained enhancers exhibit weaker H3K27ac signals compared to the HGEs and conserved enhancers (Fig. 7A) as, according to our DLM prediction, they could largely be activated by single-nucleotide mutations that potentially create binding sites of TFs active in the developing brain (Fig. 5, D and E). Therefore, we expect the activity of de novo gained enhancers to be more vulnerable to single-nucleotide substitutions. A previous massively parallel reporter assay (MPRA) in human neural stem cells targeted HGEs to identify the HGEs whose enhancer activity is substantially altered by single-nucleotide substitutions (termed hSubs) (*40*). We found that among the HGEs, those which we identified as de novo gained enhancers were more likely to harbor an hSub (Fig. 7B and Methods). Next, we aimed to assess in vivo functionality of de novo gained enhancers. On the basis of experimentally assayed developing brain enhancers from the VISTA Enhancer Browser (*15*), we found that a significantly larger fraction of de novo enhancers is active in the brain during the embryonic development (57%) as compared to HGE enhancers (15%; Fig. 7C). Again, consistent with the overall trend (Fig. 7A), the de novo VISTA enhancers exhibit weaker H3K27ac signals compared to HGE VISTA enhancers (fig. S13). Figure 7D shows three examples of de novo enhancers active in the developing human brain: *hs1686* (de novo: chr15:94525987-94526987), *hs1322* (de novo: chr1:198265687-198266687), and *hs1139* (de novo: chr1:39249975-39250975). The de novo gained enhancers are more likely to turn on the expression of a gene in human compared to the HGEs (Fig. 7E and fig. S14A), as the macaque counterpart of the human de novo gained enhancers is inactive in embryonic neocortex, whereas the macaque counterpart of HGEs is also an active enhancer, albeit relatively weaker. Therefore, all else being equal, the macaque orthologs of human genes associated with de novo gained enhancers are more likely to be silent. We also observed that, overall, the de novo enhancers are more likely to be human specific relative to chimp based on our DLM predictions (fig. S11B).

In addition, HGEs were previously shown to be associated with brain morphology–related functions such as specific functions in neuronal proliferation, migration, and cortical map organization (*13*), which differs notably from our findings, which implicate human de novo gained enhancers specifically in human neocortex development. Furthermore, Gene Ontology (GO) enrichment analysis based on either nearby genes (fig. S15) or genes linked via Hi-C contacts (tables S1 and S12) consistently shows that de novo gained enhancers are more likely to be associated with more tissue-specific functions of the developing human brain compared to HGEs (Supplementary Results 3). De novo gained enhancers are more likely to reside near (Fig. 7F) or at three-dimensional contact positions (fig. S14B) with the most tissue-specific genes in embryonic neocortex (table S6).

## DISCUSSION

Higher cognition in humans is attributed to substantial expansion of the cortical surface and increased complexity of cortical connections during early development. These phenotypic changes are likely to be mediated, in substantial part, by changes in transcriptional regulation during brain development (*1*). Recent availability of genome sequencing and epigenomic data in the developing brain of humans and a close relative—rhesus macaque—has opened the possibility to probe key regulatory changes underlying the cognitive innovations in humans. Although evolution of transcriptional regulation can be mediated by both cis- and trans-regulatory changes, our focus here is on the cis-regulatory enhancers. In contrast to previous studies of this subject (*11*–*13*, *40*), our integration of comparative epigenetic data with enhancer DLM offers several advantages. First, by integrating the estimated enhancer activity with the epigenetic marks of enhancer activity, the DLM serves as an additional filter against epigenetic noise. Second, by further incorporating predicted enhancer activity in reconstructed human-macaque ancestral genome, DLM enables us to differentiate the enhancer gains in human from the loss in macaque. Third, and importantly, the ability of DLM to predict enhancer activity changes by single-nucleotide alterations (Fig. 4A and Supplementary Results 2) enabled us to prioritize and identify the essential single-nucleotide mutations that are likely to create an enhancer in human. We demonstrate that this path of rapid enhancer gains via a single mutation might have been widely used during human brain evolution.

Although enhancers are largely active due to combinatorial binding of TFs (*10*), it is possible for a single mutation affecting a specific binding site to shift the balance in a physiologically meaningful way. A high-throughput mouse mutagenesis reporter assay based on a limb-specific enhancer (*ZRS* enhancer) showed that 71% (15 of 21) of previously published pathogenic single-nucleotide mutations at that enhancer resulted in a quantifiable change in the reporter gene expression in a pattern consistent with their pathogenic role (*41*). In addition, multiple previous studies have shown that a single mutation can create an enhancer and lead to a phenotypic change (*41*–*44*). The essential mutations are more likely to affect the chromatin accessibility (fig. S9) and therefore affect TF binding, which could explain their ability to create an enhancer. Our results suggest that single-nucleotide mutation in the human lineage, by creating binding sites for key TFs, may have induced enhancers that, mediated by a core regulatory network, involving POU3F2, PITX2, TBX20, and PITX1, underlie an increased expression in the developing neocortex of key genes involved in gliogenesis, neural tube development, and neuron differentiation. While these conclusions are based on computational analysis and should be considered preliminary, they provide a compelling resource for future experimental studies.

On the other hand, besides these essential genetic changes in human, we cannot exclude other potential drivers for de novo enhancer genesis, such as evolution of TFs and chromatin modifiers, or changes in cellular composition in the human cortex (*13*). Furthermore, analysis of single-cell RNA sequencing data from the developing human brain shows that the de novo gained enhancers are likely to be active specifically in the progenitor cells and interneurons, which, notably, are thought to underlie the expansion of the cortical surface and connectivity in the human neocortex, respectively. Given that corticogenesis in human differs from other species mainly with respect to an increased duration of neurogenesis, increases in the number and diversity of progenitors, introduction of new connections among functional areas, and modification of neuronal migration (*9*, *45*), our results are highly suggestive of a mechanistic link between de novo enhancer gains and higher cognition in humans. We also find that the de novo mutations in autistic individuals are especially enriched in the de novo gained enhancers associated with transcription activator activities, suggesting a shared basis between human cognition and autism.

Our de novo gained enhancers differ conceptually and substantively from previously identified HGEs (*13*) in terms of various functional properties. Unlike HGEs that are enhancers with increased activities in human, the de novo gained enhancers appeared to arise from neutral sequences in macaque and human-macaque common ancestor, largely attributable to single-nucleotide mutations. Therefore, the de novo gained enhancers are essentially weak enhancers created by one or a small number of TF binding site gains through single-nucleotide mutations, which explains why the de novo enhancers are more sensitive to single-nucleotide mutations (Fig. 7B). Although having weaker enhancer activity, the de novo gained enhancers are more likely to be functional embryonic human brain–specific enhancers, based on not only the functional analysis but also VISTA experimental enhancer validation (Fig. 7, fig. S15, and Supplementary Results 3). Previous studies have implicated weaker enhancers to be specifically critical during development (*46*), further suggesting a link between de novo gained enhancers and brain development.

## METHODS

### Data availability

We downloaded the gene expression data in the prenatal neocortex of human and macaque from the study (*26*), in which the homologous brain regions were identified using anatomical landmarks provided in the macaque brain atlas (*47*), and the Translating Time model (*48*) was applied to identify equivalent time points between macaque and human prenatal development (*26*). For the human analysis, we chose the 8 p.c.w. and 12 p.c.w. time points; for the macaque analysis, we selected the matching time points E60 and E82, respectively (table S13). The data are shared by the authors at http://evolution.psychencode.org/#. For a gene, we took its average expression across human individuals and time points in the neocortex tissue (NCX) to estimate its expression in human. Similarly, we took its average expression across macaque individuals and approximately matching time points in the tissue of NCX to estimate its expression in macaque. The sequence of common ancestor of human and macaque were obtained from the study (*14*). The single-cell transcriptomic data of developing human neocortex during midgestation (*17*) are shared by the authors at http://solo.bmap.ucla.edu/shiny/webapp/. In addition to assigning enhancers to their nearby genes, we also used the Hi-C loops from midgestation developing human cerebral cortex (*49*) to link enhancers to their gene targets. As the Hi-C experiments were conducted separately in two major zones of neocortex, germinal zone (GZ) and cortical plate (CP), and de novo gained enhancers are largely active in progenitor cells and interneurons, which, in turn, are both enriched in GZ (*50*), we use the GZ Hi-C loops to link enhancers to their target genes. The CS23 HGEs and H3K27ac signals were obtained from the study (*13*). All the potential fetal brain enhancers [the merged Assay for Transposase-Accessible Chromatin with high-throughput sequencing (ATAC-seq) peaks from the GZ and CP of the human developmental brain] were obtained from the study (*51*). The fetal brain eQTLs were obtained from the study (*16*). The HepG2 regulatory activity QTL (raQTL) data were obtained from (*52*). For the allelic imbalance analysis, we obtained H3K27ac data of human embryonic neocortex (CS23, p.c.w. 8.5) (*13*) from http://noonan.ycga.yale.edu/, and DHS data (ENCSR595CSH, at approximate developmental time points E56 and E58) from (*18*, *19*).

### Embryonic neocortex enhancers in human, rhesus macaque, and mouse

The H3K27ac peaks of all three species were obtained from a previous study (*13*). The enhancers were defined as H3K27ac peaks extended to 1 kb from its original center. Integrating wider sequence context is critical because sequence surrounding the variant position determines the regulatory properties of the variant, as in vivo TF binding depends upon sequence beyond traditionally defined motifs (*53*, *54*). Enhancers overlapping promoters (including all alternative promoters) and promoters (intervals [−1000 base pairs (bp), 1000 bp] surrounding the transcription start site) were removed from the enhancer set. Overall, we identified 32,201 human enhancers, 43,997 macaque enhancers, and 43,155 mouse enhancers. The developmental stage and cell type matching between species was done through careful examination by the authors of the original study (*13*).

### A deep convolutional neural network model for enhancer prediction

We built a deep convolutional neural network to predict tissue-specific enhancer activity directly from the enhancer DNA sequence. The DLM comprises five convolution layers with 320, 320, 240, 240, and 480 kernels, respectively (table S14). Higher-level convolution layers receive input from larger genomic ranges and are able to represent more complex patterns than the lower layers. The convolutional layers are followed by a fully connected layer with 180 neurons, integrating the information from the full length of 1000-bp sequence. In total, the DLM has 3,631,401 trainable parameters. We used the Python library Keras version 2.4.0 (https://github.com/keras-team/keras) to implement our model.

The model was trained for each of the four temporal-spatial groups of enhancers (CS16, CS23, F2F, and F2O). The positive sets contain the human embryonic enhancers of each group. The DHS profiles of non–CNS-related and nonembryonic tissues from Roadmap Epigenomics projects (*55*), which do not overlap the positive sets, were collected as the negative training set of the DL model. The reason we used DHS sites not overlapping embryonic neocortex H3K27ac peaks as negative control regions is that we aim to identify tissue-specific enhancers of embryonic neocortex, and DHS is a good representation of active chromatin. The fact that DHS in general overlaps H3K27ac makes it a stringent control, and in fact, our choice of DHS as the control is analogous to DeepSEA, which uses the genomic regions not overlapping the positive set and with at least one TF binding as the negative set, which broadly overlap with DHS regions.

Training and testing sets were split by chromosomes. Chromosomes 8 and 9 were excluded from training to test prediction performances. Chromosome 6 was used as the validation set, and the rest of the autosomes were used for training. Each training sample consists of a 1000-bp sequence (and their reverse complement) from the human GRCh37 (hg19) reference genome. Larger DL score of the genomic sequence corresponds to a higher propensity to be an active enhancer. The genomic sequence with DLM score ≥ 0.197 (FPR ≤ 0.1) is predicted to be active enhancers. We used the difference of the DLM score induced by a human-macaque single-nucleotide mutation to estimate its impact on enhancer activity.

Given a human (hg19) or macaque (rheMac2) enhancer, we used liftOver (*56*) to identify their orthologs. Only the reciprocal counterparts with their length difference no more than 50 bp were considered to be ortholog pairs. For a human sequence with *n* mutations relative to its macaque ortholog, to score the impact of combinations of *m* (*m* < *n*) mutations on enhancer activity, all possible combinations of *m* (*n* choose *m*) human alleles at the human-macaque mutation sites were introduced to the macaque orthologs if the total number of combinations (*n* choose *m*) is no more than 10,000; otherwise, we randomly sample 10,000 combinations of *m* human alleles from the human-macaque mutation sites and introduce them to the macaque ortholog. The change of DL score caused by the set of introduced human mutations was used to estimate their impact on enhancer activity.

We applied the same convolutional neural network architecture to build a HepG2 enhancer (H3K27ac peaks centered by DNase peaks) classifier. Next, we further used the HepG2 DLM to evaluate the allele-specific effects on enhancer activity using raQTLs (*52*).

### Gain and loss of enhancers

Briefly, if a human enhancer with a high DLM score scored low both in macaque and in the common ancestor, and was not detected by H3K27ac in macaque, it was considered to be a de novo gain in humans (Fig. 1A). Likewise, if a macaque enhancer with a high DL score scored high in common ancestor, scored low in human, and was undetectable by H3K27ac in human, it was considered a loss in human (Fig. 1A). The enhancers that are detected by H3K27ac in both human and macaque and scored highly in all three genomes were called conserved enhancers (Fig. 1A).

### Normalization of gene expression data

We applied “tmm” built-in normalization method of edgeR to normalize human and macaque embryonic neocortex gene expression and to remove differences across species and batch effects. To identify the most tissue-specific genes of human embryonic neocortex, the expression data of human individuals were averaged and quantile-normalized together with the gene expression profile downloaded from GTEx. The top 2000 genes with the highest ratios of the human embryonic expression to the mean of the GTEx expression were identified as the most specifically highly expressed genes in human embryonic neocortex (table S6).

### De novo single-nucleotide substitutions in ASD

We obtained 127,141 de novo single-nucleotide mutations in ASD from a previous study (*39*), which were identified from Simons Simplex Collection of whole-genome sequencing data for 1790 families that were available via the Simons Foundation Autism Research Initiative (SFARI).

### Functional enrichment analysis using GREAT and DAVID tools

To probe the potential functional roles of gained and lost enhancers, we first tested for functional enrichment among genes near the enhancer loci using the online Genomic Regions Enrichment of Annotations Tool (GREAT) version 3.0.0 (*57*) using single nearest gene association rule with more strict settings than default. Specifically, the GO terms will be considered as enriched if it has at least 10 gene hits with false discovery rate (FDR) threshold set as 0.01. Two background options were used when using GREAT. Figure 2 (D and E) and figs. S5 and S15 are based on enrichment against whole genome region. Next, we performed GO enrichment analysis using all potential fetal brain enhancers (the merged ATAC-seq peaks from GZ and CP of the human developmental brain) (*51*) as the background and obtained consistent observations (figs. S4, A and B, and S16, A and B). The exception is the conserved enhancers, which are not enriched for CNS-related biological processes (fig. S16A). The tissue-specific signal of conserved enhancers is dampened, as expected, by using the fetal brain enhancers as the background, as the conserved enhancers constitute most of the fetal brain enhancers. We also applied DAVID (*58*, *59*) to do functional enrichment of the genes with Hi-C loops to different sets of enhancers.

### Enrichment analysis of GWAS traits and eQTLs

The NHGRI-EBI GWAS Catalog (*60*) was downloaded. To study the enrichment of a set of SNPs coinciding with CNS-related GWAS traits, the tag SNPs were first expanded by LD (*r*^2^ > 0.8, maximum distance of 500 kb) using Plink [https://zzz.bwh.harvard.edu/plink/ld.shtml; (*61*)] with the following parameters: *“--r2 --ld-window-kb 500 --ld-window-r2 0.8*.”

We overlapped the LD-expanded GWAS traits with the human-macaque mutation sites of the gained enhancers where the human alternative alleles are the same as the macaque reference alleles. The CNS-related GWAS traits are listed in tables S4 to S6. We then use the fraction of CNS-related traits among the total GWAS traits overlapping the essential mutations, as compared to that of the nonessential mutations to estimate the enrichment of CNS-related traits in the essential mutation positions (Fig. 5A).

As for the overall enrichment of the CNS-related GWAS traits in the three sets of enhancers (Fig. 2F), we used the density (average number of LD-expanded GWAS traits per enhancer) to estimate the enrichment. As the density of common SNPs in the three sets of enhancers (average number of SNPs per enhancer) is comparable (gained: 4.1, lost: 4.05, conserved: 4.6) and would not change the trend of the enrichment upon normalization, we did not normalize the GWAS density by SNP density.

As for the enrichment of the fetal brain eQTLs (*16*) in the three sets of enhancers, we first compared the density of eQTLs (average number of eQTLs per enhancer) in the three sets of enhancers (Fig. 2C). Next, we normalized the fraction of eQTLs fallen within a set of enhancers by the fraction of common SNPs fallen within that set of enhancers (fig. S3).

### Identification of potential TFBSs in the de novo gained enhancers

To identify potential binding sites, we used FIMO (*62*) to scan the profiles of binding sites for vertebrate TF motifs in Jaspar (*63*), CIS-BP (*64*), SwissRegulon (*65*), HOCOMOCO (*66*), and UniPROBE (*67*) databases, along the enhancer sequences. We identified motif-specific thresholds to limit the FDR to no more than five false positives in 10 kb of sequence, by scanning each motif on random genomic sequences using FIMO (*62*). Enrichment of a motif in de novo gained (foreground) relative to conserved (background) enhancers was ascertained using Fisher’s exact test. The occurrence of a particular Transcription Factor Binding Site (TFBS) in the set of de novo gained/conserved sequences was normalized by the total number of de novo gained/conserved regions.

However, when identifying TFs whose motifs are enriched in de novo gained enhancers relative to conserved enhancers, we included both the human and the macaque ortholog sequences, to avoid allelic bias in our following analysis of activation/repression of enhancers by single-nucleotide mutations. Next, we assessed whether a mutation (in a de novo gained enhancer) creates a binding site of a potential activator or disrupts binding of a potential repressor. We estimated, for each enriched TF, the ratio of binding site gain to loss caused by essential mutations within de novo gained enhancers relative to the same ratio caused by common SNPs. If the gain/loss (loss/gain, respectively) ratio caused by essential mutations was greater than 1.2-fold that for common SNPs, the TF was considered activator (repressor, respectively).

### Identification of allelic imbalance in H3K27ac and DHS data

We reasoned that if an essential mutation locus happens to be heterozygous in a sample, we would expect the two alleles to have differential enhancer activity (specifically, the derived allele should have higher enhancer activity than the ancestral allele), which should be reflected in the differential representation of the two alleles among the H3K27Ac reads of the locus. We used BWA (*68*) to map two replicates of CS23 H3K27ac data (*13*) to hg19 human reference sequence. At the mutation/SNP sites, the H3K27ac reads were extracted using BaalChIP (*69*). Allelic counts over heterozygous sites of the two replicates were merged, and variants that had at least six reads were further processed for allele-specific enhancer activity analysis with binomial test. We use the heterozygous sites within the activity preserved enhancers (the ratio between human and macaque H3K27ac signal is no more than 1.2) as the background. For a heterozygous site, if the ratio of read number of the human allele to that of the macaque allele is over 1.3 and the binomial *P* value ≤ 1 × 10^−3^, the position is considered to have allelic imbalance. As for the allelic imbalance in DHS data, we obtained the DHS reads mapped to hg19 (bam files) from ENCSR595CSH (similar developmental time points E56 and E58) and applied the same procedure to identify allelic imbalance at heterozygous sites. Notably, the essential mutations are identified based on the DLM, based on the indication that the human alleles at these positions are likely to create enhancers. The human alleles at these essential positions coincidently (and independently) exhibit more H3K27ac reads than do the macaque alleles (termed allelic imbalance), substantiating that the human alleles are associated with increased enhancer activity.

### Logistic regression

We applied the “glm” method from the R package to apply logistic regression to predict gene expression change (human – macaque) using categories of de novo gained enhancers (with essential mutations, with nonessential mutations, with common SNPs) and gene expression in macaque.

$$\mathrm{glm}(\text{Δ}g∼\text{de novo enhancer category}+g^{\mathrm{macaque}},\mathrm{family}={}^{\prime }{\mathrm{binomial}}^{\prime })$$

where ∆*g* is the gene expression change in human compared to macaque (*g*^human^ − *g*^macaque^), which is binary (1 for positive change, 0 for negative change); de novo enhancer category is binary for each category (de novo enhancer with essential mutations, de novo enhancer with nonessential mutations, de novo enhancer with common SNPs); and *g*^macaque^ is the expression level of the gene in macaque. Positive coefficient of each variable indicates positive correlation between the variable and ∆*g*.

### Single-cell clustering and visualization

Clustering was performed using Seurat (v2.3.4) (*70*). Read depth normalized expression values were mean-centered and variance-scaled for each gene, and the effects of number of unique molecular identifier (UMI) (sequencing depth), donor, and library preparation batch were removed using a linear model with Seurat (“ScaleData” function). Highly variable genes were then identified and used for the subsequent analysis (Seurat “MeanVarPlot” function). Briefly, average expression and dispersion are calculated for each gene, genes are placed into bins, and then a *z* score for dispersion within each bin is determined. Principal components analysis (PCA) was then used to reduce dimensionality of the dataset to the top 13 PCs (Seurat “RunPCA” function). Clustering was then performed using graph-based clustering implemented by Seurat (“FindClusters” function). Cell clusters with fewer than 30 cells were omitted from further analysis. Clusters were annotated using the Seurat function “group.by.”

For visualization, t-distributed stochastic neighbor embedding (tSNE) coordinates were calculated in PCA space, independent of the clustering, using Seurat (“RunTSNE” function). tSNE plots were then colored by the cluster assignments derived above, gene expression values, or other features of interest. Gene expression values are mean-centered and variance-scaled unless otherwise noted.

### DoS test

The DoS test was designed to measure the direction and extent of departure from neutral selection based on the difference between the proportion of substitution and polymorphism in the selective sites. DoS is positive when there is evidence of adaptive evolution, is zero if there is only neutral evolution, and is negative when there are slightly deleterious mutations segregating (*20*). Here, we used the mutated fourfold degenerate sites as the background to measure the selection on the mutations within de novo gained enhancers (Eq. 1). Note that all sites in our three mutational site classes are, by design, mutated in human relative to macaque. Therefore, to avoid ascertainment bias, we uniformly applied the same criteria of human-macaque mutation to select a subset of all fourfold degenerate sites.

Let *n* represent the “nonsynonymous” sites, i.e., the essential or nonessential mutations within the de novo gained enhancers. *S* represents the nonsynonymous sites, i.e., the mutated fourfold degenerate sites. *D* means “diverged” sites, i.e., mutations (or substitutions) that are fixed in the human populations, and *P* means “polymorphic” sites, i.e., both the ancestor allele and the mutations are preserved in the human populations (Table 1).

DoS=Dn/(Dn+Ds)−Pn/(Pn+Ps) (1)

**Table 1.** Contingency table of number of fixed mutations and polymorphic mutations at the foreground and background sites. *Ds*, number of fixed mutations at mutated fourfold degenerate sites; *Dn*, number of fixed mutations within de novo gained enhancers; *Ps*, number of polymorphic mutations at mutated fourfold degenerate sites; *Pn*, number of polymorphic mutations within de novo gained enhancers.

	Fixed	Polymorphic
Mutated fourfold degenerate sites	*Ds*	*Ps*
Mutated sites within gained enhancers	*Dn*	*Pn*

### Comparing de novo gained enhancers and HGEs using MPRA data

Overlapping the essential human substitutions (relative to chimp, termed hSubs) from a MPRA targeted HGEs (*40*) with the de novo gained enhancers, we found that 141 de novo gained enhancers overlapping HGEs (dubbed de novo HGEs) were tested by this assay. In total, 14 of the 141 (10%) de novo HGEs harbor at least one hSub. For the 1019 CS23 HGEs that do not overlap de novo gained enhancers (dubbed non–de novo HGEs), 74 (7%) HGEs have at least one hSubs (Fig. 7B). We applied 90% bootstrapping 50 times to estimate the statistical significance of the difference between the two fractions (Fig. 7B).

## Notes

View/request a protocol for this paper from *Bio-protocol*.
