# Supplementary material for: De novo human brain enhancers created by single-nucleotide mutations
Source: Sci Adv. 2023 Feb 15;9(7):eadd2911. doi: 10.1126/sciadv.add2911 (PMC9931207; doi:10.1126/sciadv.add2911)
Supplement: Supplementary file 1 — Supplementary Text Figs. S1 to S22 [file sciadv.add2911_sm.pdf]

Science Advances
AAAS

Supplementary Materials for

De novo human brain enhancers created by single nucleotide mutations

Shan Li *et al.*

Corresponding author: Ivan Ovcharenko, ovcharen@nih.gov; Sridhar Hannenhalli, sridhar.hannenhalli@nih.gov

Sci. Adv. **9**, eadd2911 (2023)
DOI: 10.1126/sciadv.add2911

The PDF file includes:

Supplementary Text
Figs. S1 to S22

Other Supplementary Material for this manuscript includes the following:

Data S1

Supplementary Text

Supplementary Results

1. Performance and further validation of DLM of embryonic neocortex enhancers

Here we provide benchmarking and comparison of our enhancer model with DeepSEA (*71*).

To directly compare our model performance with DeepSEA, we applied our model to the training and testing H3K27ac data sets used by DeepSEA. Our model achieved a very similar (although slightly higher) accuracy (both auROC and auPRC) compared to DeepSEA across multiple datasets (Figure S17AB).

We have shown that the human embryonic neocortex DLM can accurately estimate the enhancer activity (independently) in macaque from its genomic sequence (Fig 2A). To further validate our model, we applied the model trained on the human embryonic neocortex (CS23) enhancers (H3K27ac peaks) (*13*) and tested it on the macaque embryonic neocortex enhancers (H3K27ac peaks) (*13*) (Figure S17CD), using 10-fold random genomic regions (due to a lack of available multi-tissue DHS profile) that do not overlap H3K27ac peaks as the negative testing set. The model still achieves an auROC of 0.9 at e79F (Figure S17C). We also apply our DLM to the sequences of all enhancers in VISTA, and consistently observe that the brain enhancers active in human (respectively, mouse) have larger DLM scores than those that are inactive in human (respectively, mouse) brain (Figure S18).

2. DLM can accurately predict allele specific effects on histone marks H3K27ac

Our DLM is trained to distinguish enhancer region from non-enhancer regions in a specific context. However, its application to identify *de novo* enhancer gains driven by single nucleotide mutations requires the DLM score to be sensitive to single nucleotide changes. We performed additional analyses to ensure that DLM score indeed (i) represent the enhancer activity level, and (ii) is sensitive to single nucleotide changes.

First, we computed the direct correlation between the predicted DLM score (DL score) of the enhancers and the log of their average H3K27ac signal intensity. We observed a significant positive correlation between the two (correlation = 0.4, empirical p-value = 3.18e-6).

Next, DeepSEA was shown to work well in identifying variants at loci that affect histone signals (hQTLs of H3K27ac or H3K4me3) (*71*). As our approach is very similar to DeepSEA (just a different neural net architecture), and we aim to identify variants that create enhancers, we trained our model on H3K27ac peaks in a lymphoblastoid cell line, GM12878, and applied it to predict the same set of hQTLs of H3K27ac in lymphoblastoid cell lines (*72*) as did DeepSEA. Our model shows similar accuracy as DeepSEA (Figure S19).

To further show the ability of our DLM to accurately predict enhancer activity from sequence with single-nucleotide sensitivity, we first trained a deep learning enhancer (H3K27a peaks) model in HepG2 cell line and used it to predict the allele-specific effects of raQTLs (*52*) on enhancer activity, which has high accuracy (Figure 4A). Next, we applied our CS23 model to evaluate the 2,578 allelically imbalanced SNPs within the CS23 H3K27ac peaks, which were identified using the R-package BaalChIP (*69*). Our model makes similarly accurate predictions on this set of SNPs as well (Figure S20). In this study, the DLM score at FPR <= 0.1 was set as the cutoff to identify potential active enhancers. we have additionally used a more stringent threshold (FPR <= 0.05) and obtained 1,064 potential enhancers with higher DLM scores (26% of the original 4,066 enhancers). Very encouragingly, this more stringent set of enhancers exhibits stronger

signals in terms of increase in the expression of the target gene, enrichment of eQTLs and allelic imbalance at essential mutation positions (Figure S21). This analysis justifies the use of stringent FPR cutoffs for the selection of a limited set of enhancers with the most pronounced downstream effect for follow-up testing and investigation.

3. Using Hi-C loops to link enhancers to their potential target genes

In the main result sections, we opted to use proximity as the criterion to identify the enhancer-associated gene for several reasons. First, the available human Hi-C contacts (*49*) are very sparse: only 23% of human embryonic neocortex enhancers are covered. Second, in the study of 'Activity-by-Contact model' (*73*), based on a small number of experiments, the authors concluded that it is rare for an enhancer to skip the nearest gene (*73*). Finally, for the enhancers included in Hi-C loops, around 60% of *de novo* gained enhancers contact their nearest genes, and more than 50% of both lost and conserved enhancers are in contact with their nearest genes (Figure S22), suggesting that our findings based on the nearest genes are robust.

Nevertheless, we examined the results when the enhancers were mapped to their putative targets based on Hi-C loops. The findings based on the Hi-C loops are consistent with the ones based on the proximity rule. For example, the *de novo* gained enhancers tend to associate with an increase in the expression of their target gene, whereas the lost enhancers show the reverse trend (Figure 2A and Figure S2). Enhancers are more likely to regulate the tissue-specific genes of embryonic neocortex either based on proximation rule (Figure 7B) or Hi-C contacts (Figure S14B). In addition, using either gene proximation rule (Figure 7C) or Hi-C contact (Figure S14A), we observed that *de novo* gained enhancers are more likely to turn on gene expression compared to HGEs.

Supplementary Figures

Figure S1

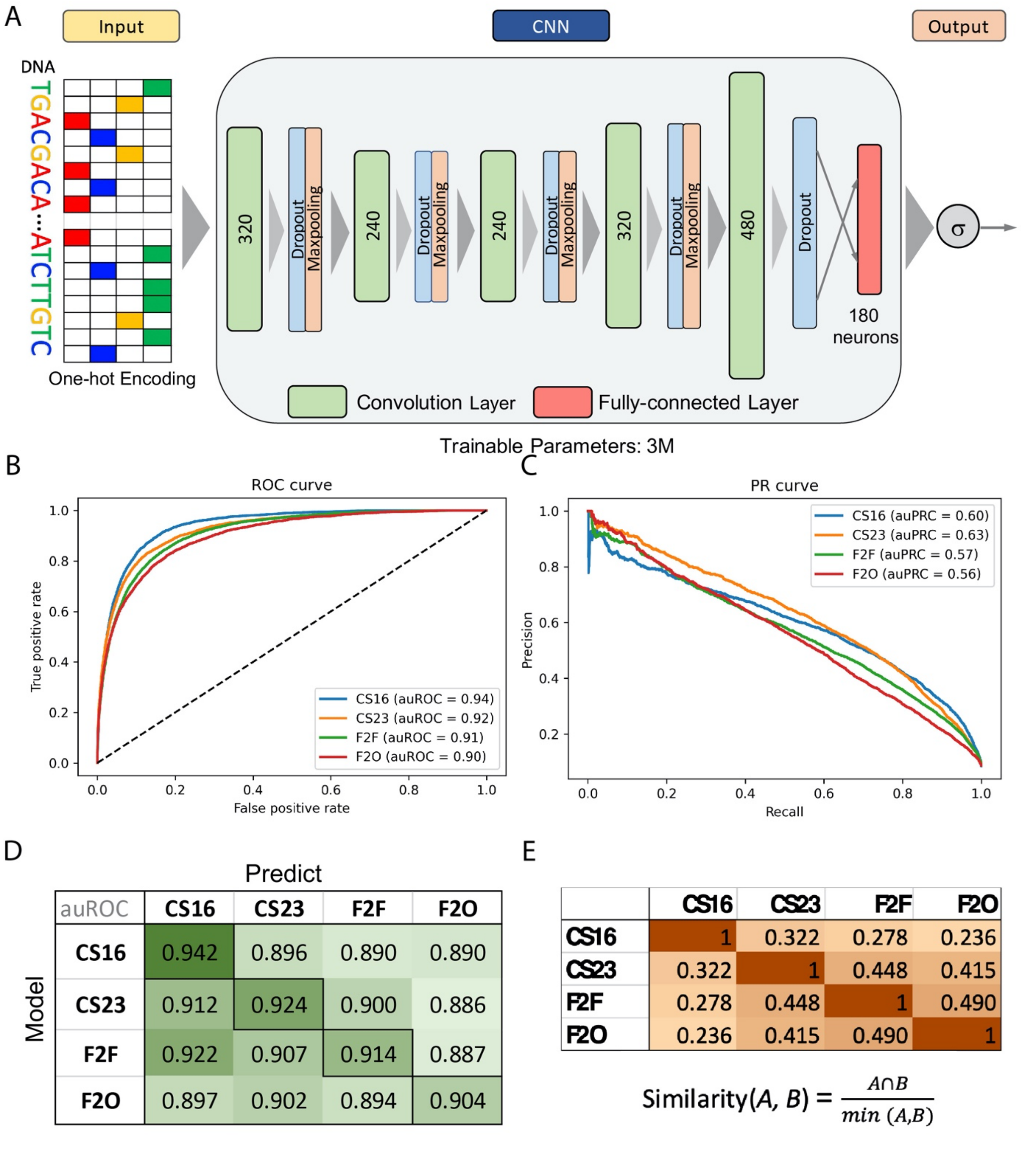

Figure S1. **Deep learning model of human embryonic neocortex enhancers used to score enhancer activity.** A) Structure of the deep convolutional model. The number within each

convolutional layer indicates the number of kernels. B) ROC curve of the model. C) PR curve of the model. D) Model performance across four stages. E) Similarity between enhancer sets across stages.

Figure S2

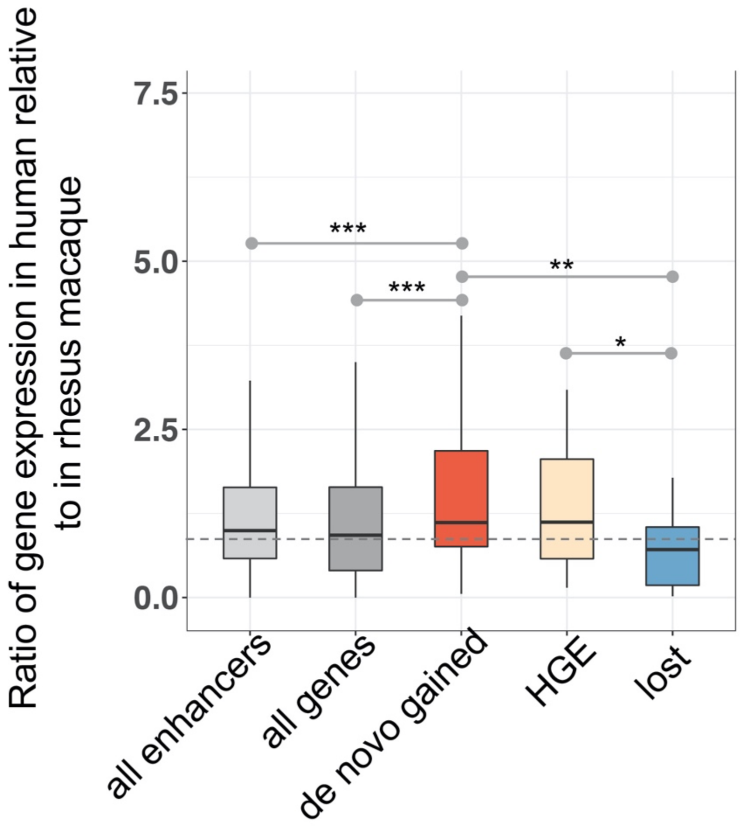

Figure S2. **The expression level of genes with Hi-C loops to the *de novo* gained enhancers is increased, and so is the previously published enhancers that increase activity in human (HGEs)** (*13*). By contrast, the genes in contact with the lost enhancers show the reverse trend. "all enhancers" refer to the genes link to all enhancers. *Wilcoxon p-value <= 0.01. ** Wilcoxon p-value <= 1e-3. *** Wilcoxon p-value <= 1e-5.

Figure S3

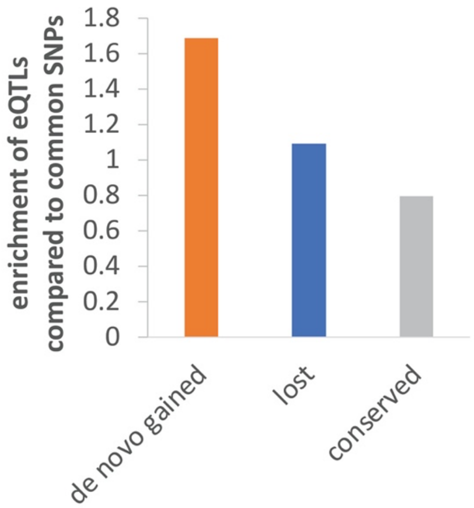

Figure S3. **Enrichment of eQTLs compared to common SNPs in the three sets of enhancers.** Specifically, the enrichment = fraction of eQTLs in enhancers/fraction of SNPs in enhancers.

Figure S4

A

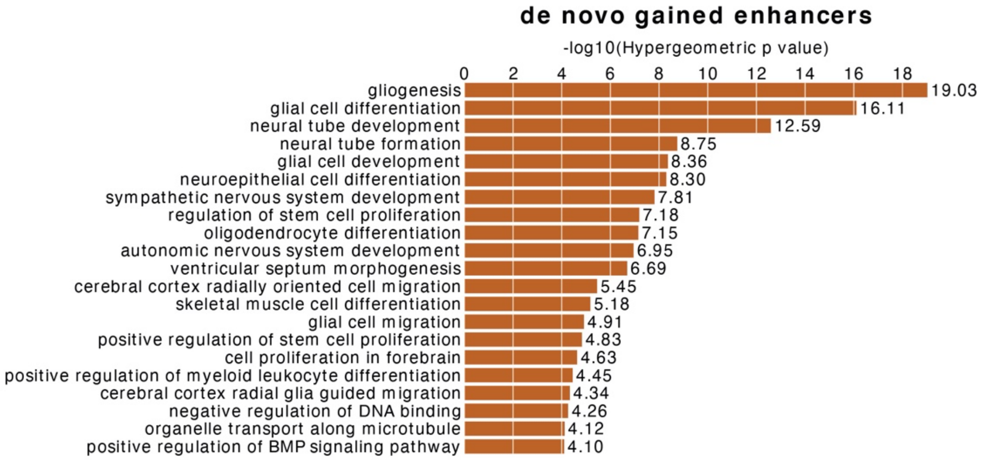

B

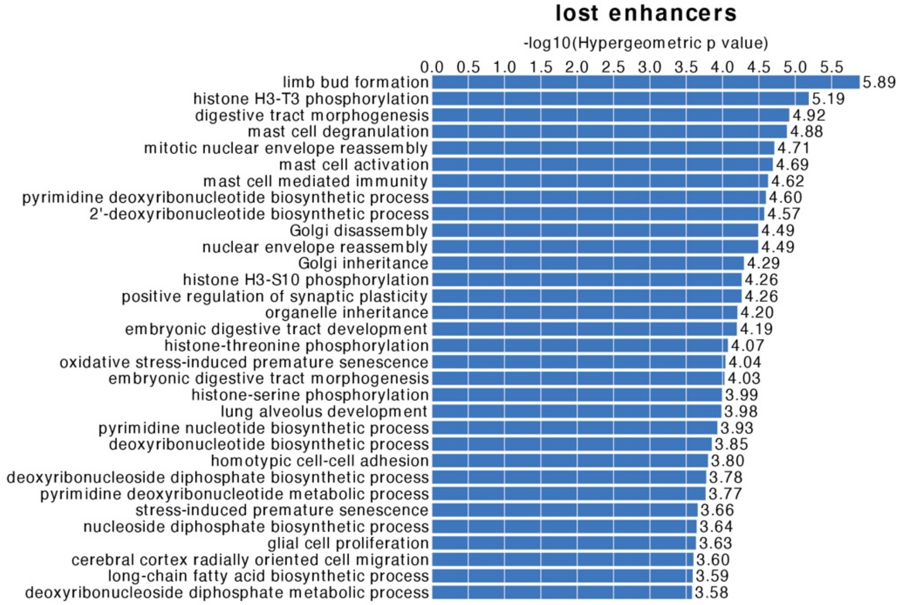

Figure S4. ***De novo* gained enhancers are associated with essential CNS-related biological processes, using all fetal brain enhancers (*51*) as the background.** (A) GO terms of *de novo* gained enhancers. (B) GO terms of lost enhancers. We apply GREAT with the single nearest gene association rule to do functional enrichment of genes near enhancers. The GO terms will be considered as enriched if it has at least 10 gene hits with FDR threshold set as 0.01.

Figure S5

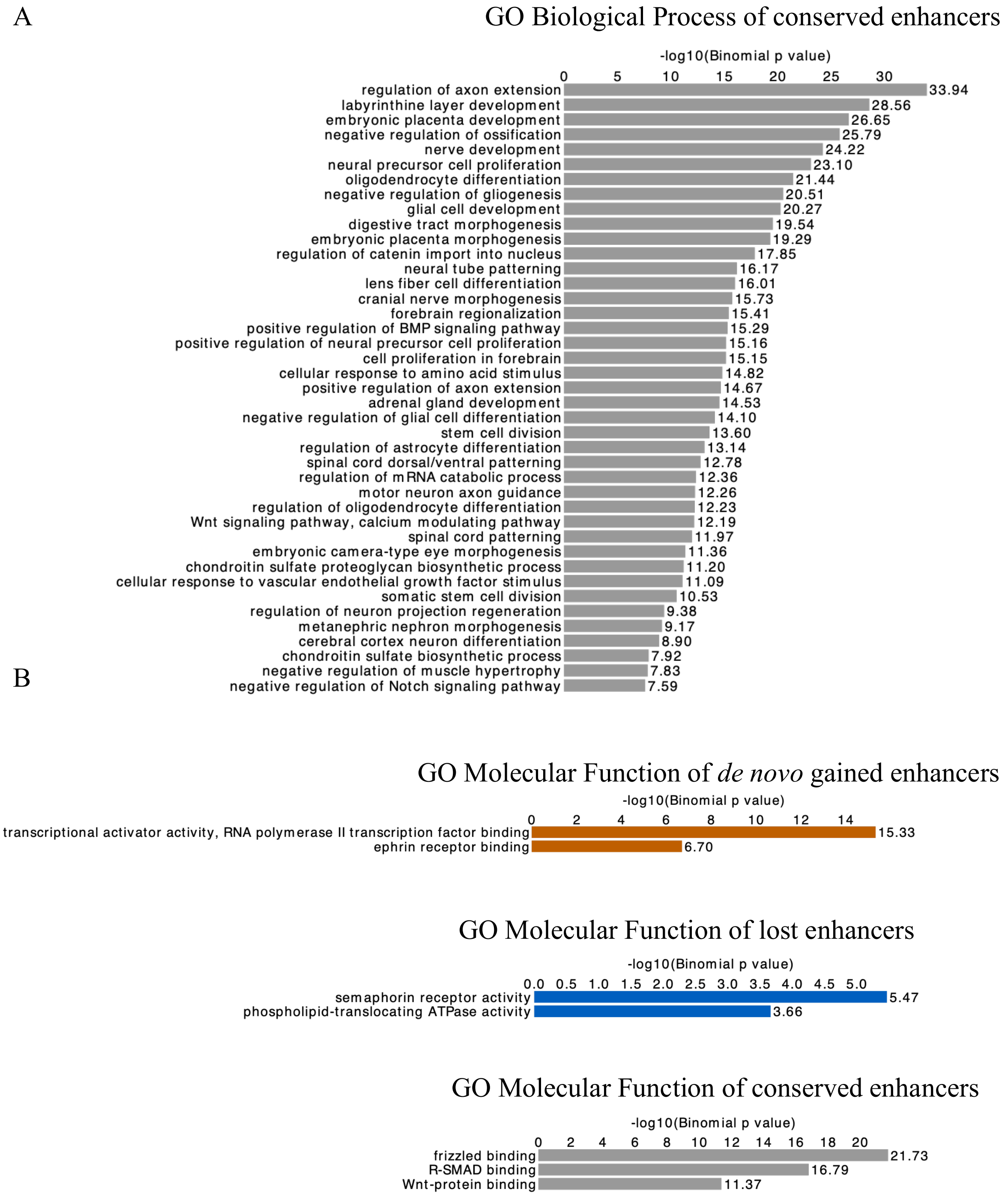

Figure S5. **GO enrichment of different sets of enhancers using whole genome as background.** A) Enriched GO Biological Processes terms of conserved enhancers. B) Enriched GO Molecular Function terms of the three sets of enhancers.

Figure S6

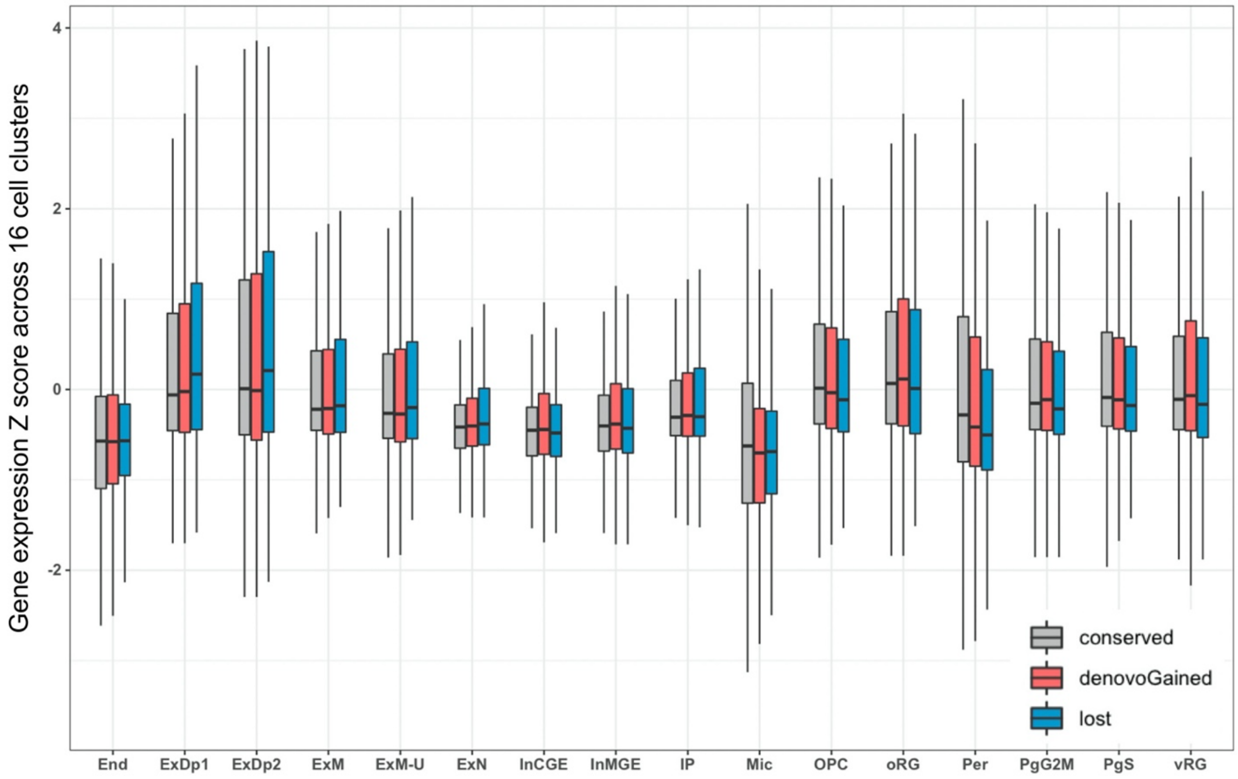

Figure S6. **Z scores of expression of genes nearby the three sets of enhancers across 16 cell clusters.** The lack of statistical significance may partly be due to the high variability/noise in single cell gene expression data, and also because only a subset of the genes near *de novo* gained enhancers are likely to drive cluster-specific expression as revealed in our fractional analysis (Figure 3C) but obscured in our analysis of z-scores for all genes.

Figure S7

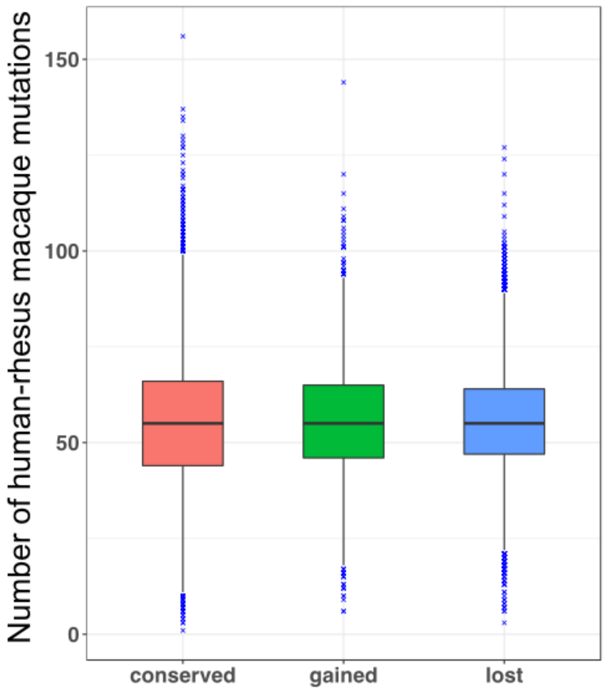

Figure S7. **Number of human-macaque mutations within enhancers.**

Figure S8

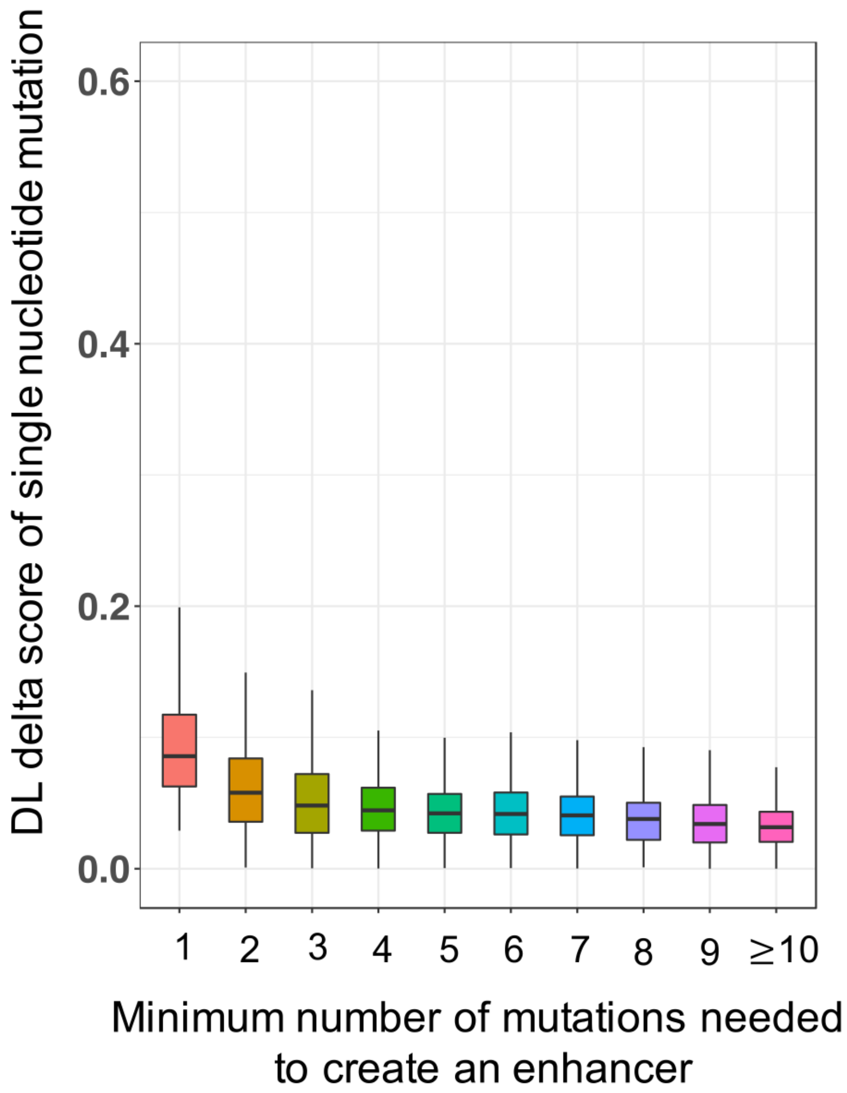

Figure S8. **Distribution of delta score of the single nucleotide mutations that are minimally needed to create an enhancer.**

Figure S9

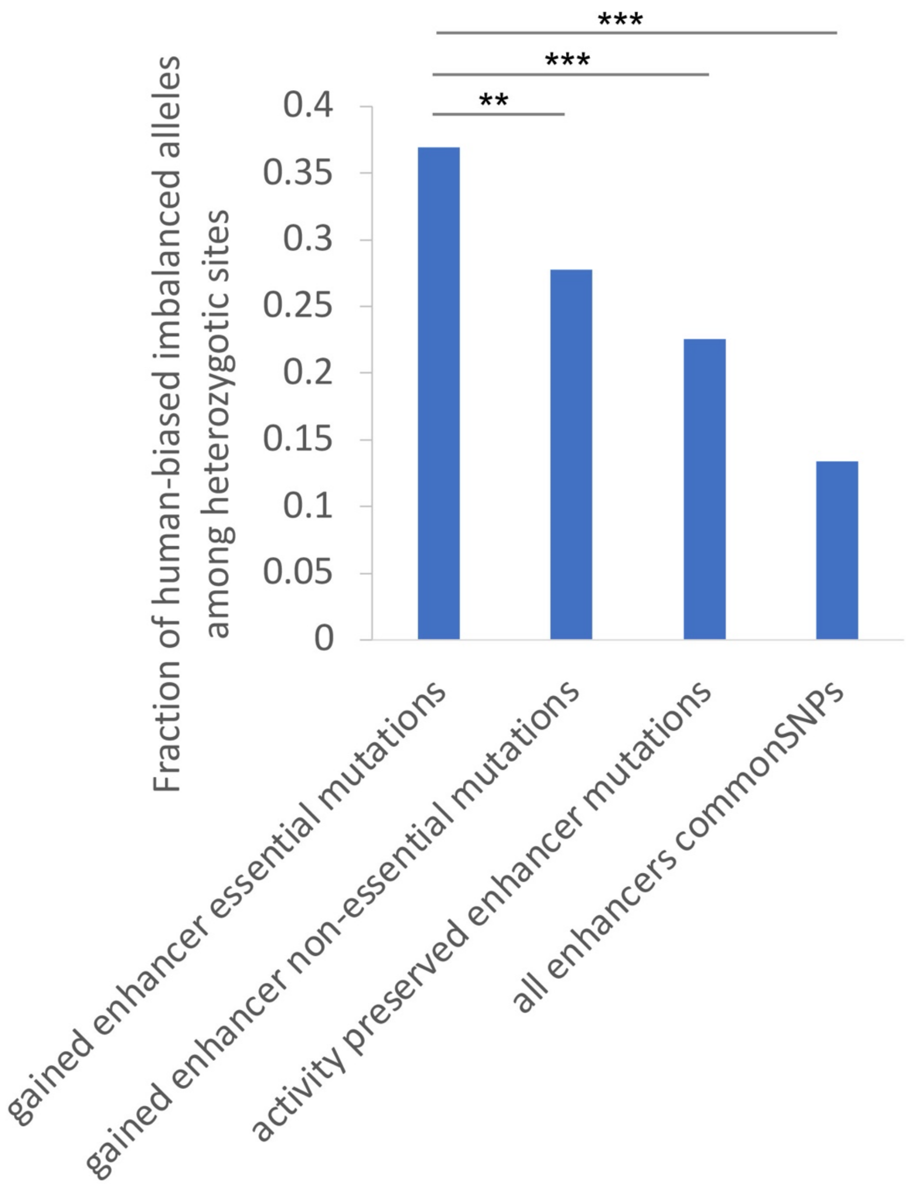

Figure S9. **Fraction of polymorphic sites with allelic imbalance for DHS reads.** ** indicates Fisher's exact test P-value <= 0.01. ** refers to Fisher's exact test P-value <= 1e-3.

Figure S10

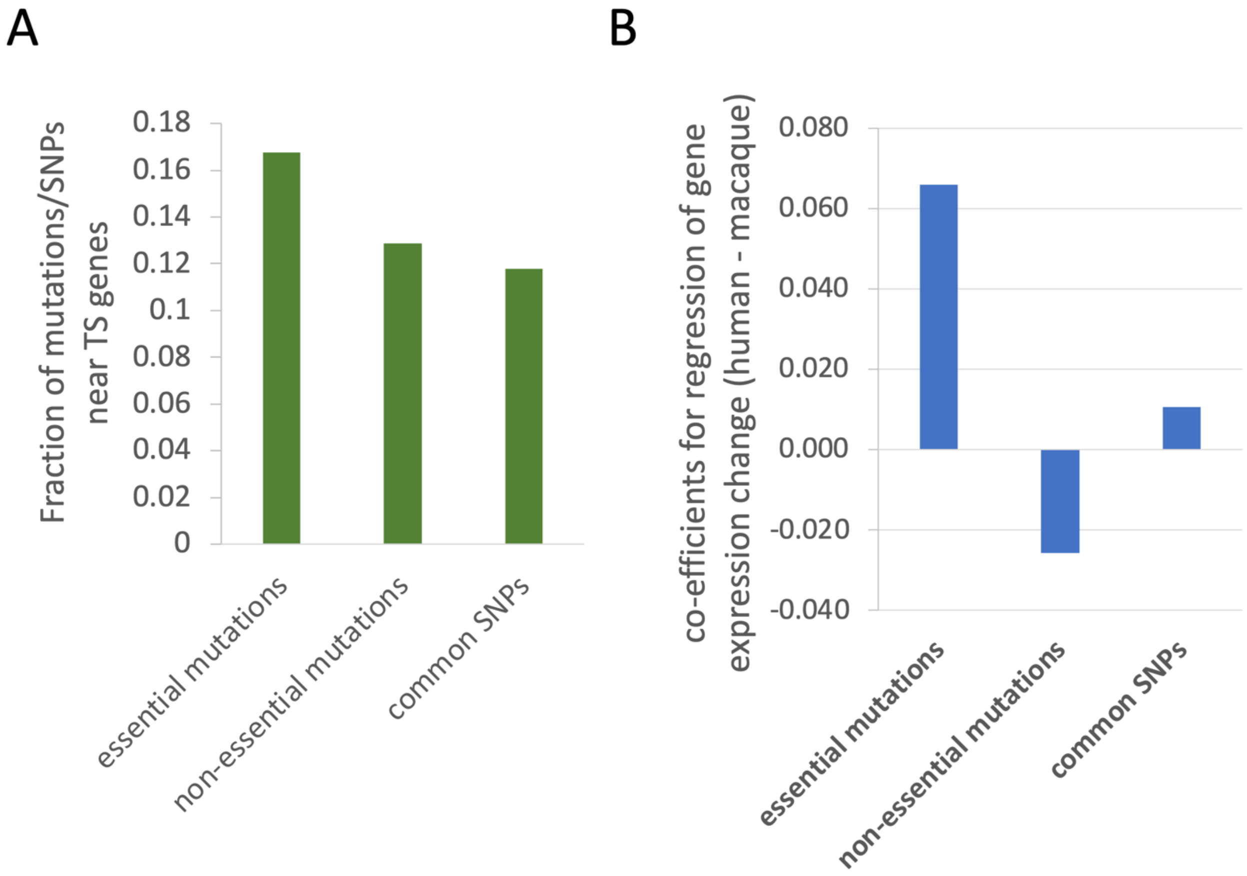

Figure S10. **Association between essential mutations and regulatory changes in genes during human embryonic neocortex development.** (A) Fraction of mutations/SNPs near TS genes (Table S6). (B) Coefficients for regression of gene expression change (human – macaque) against three categories, de novo gained enhancers with essential mutations, de novo gained enhancers with non-essential mutations, and de novo gained enhancers with common SNPs.

Figure S11

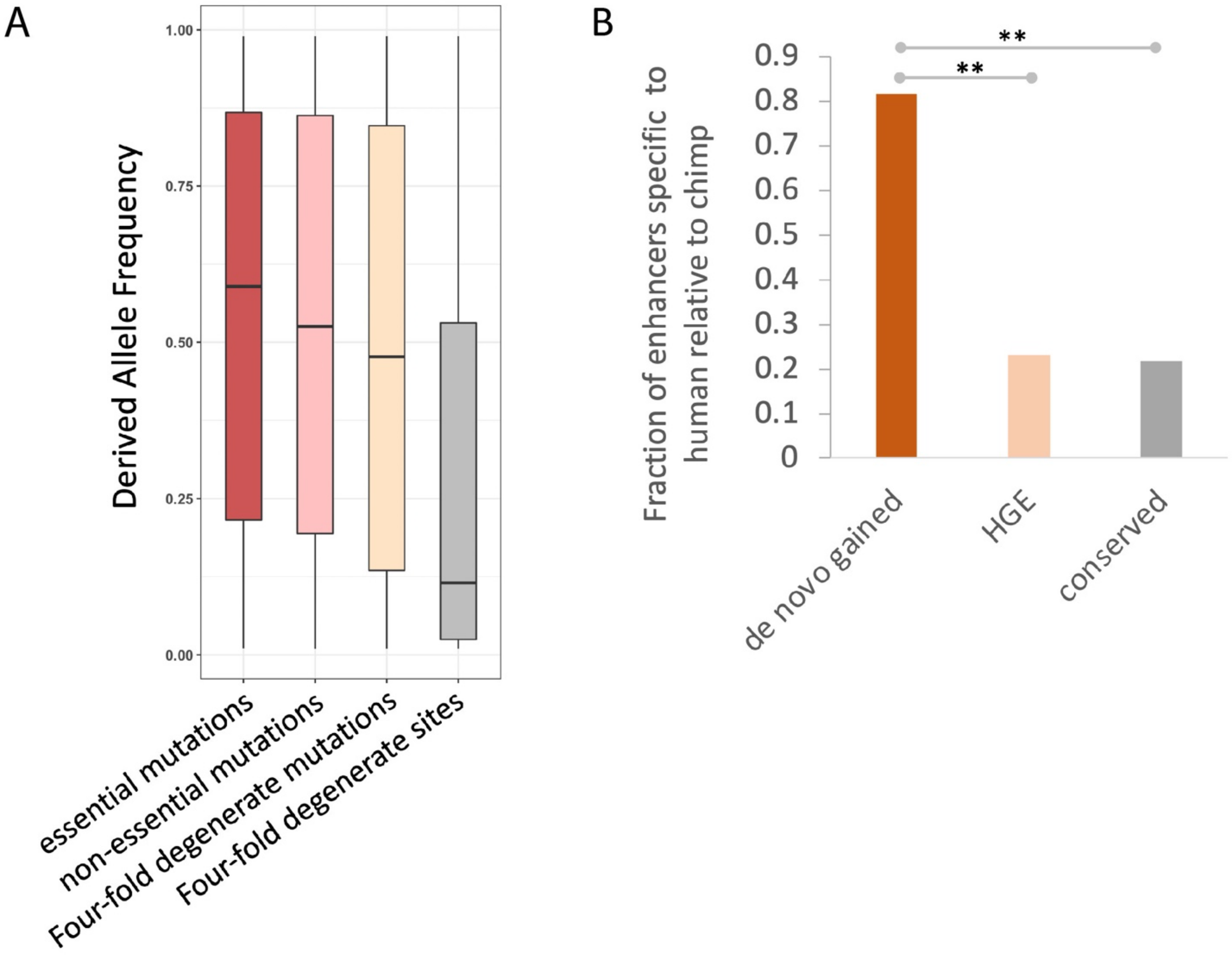

Figure S11. **Evolutionary selection on *de novo* gained enhancer and essential mutations.** (A) Derived allele frequency of polymorphic sites among the three groups of detected mutations, and four-fold degerate sites. (B) Fraction of enhancers that are specific to human, i.e., detected in human by our model but not in orthologous locus in chimp . ** refers to Fisher's exact p-values <= 1e-3. In Figure A, the wilcox p-value between essential mutations vs. non-essential mutations is smaller than 0.05. The wilcox p-value between essential mutations vs four-fold degenerate mutation sites is smaller than 1e-3.

Figure S12

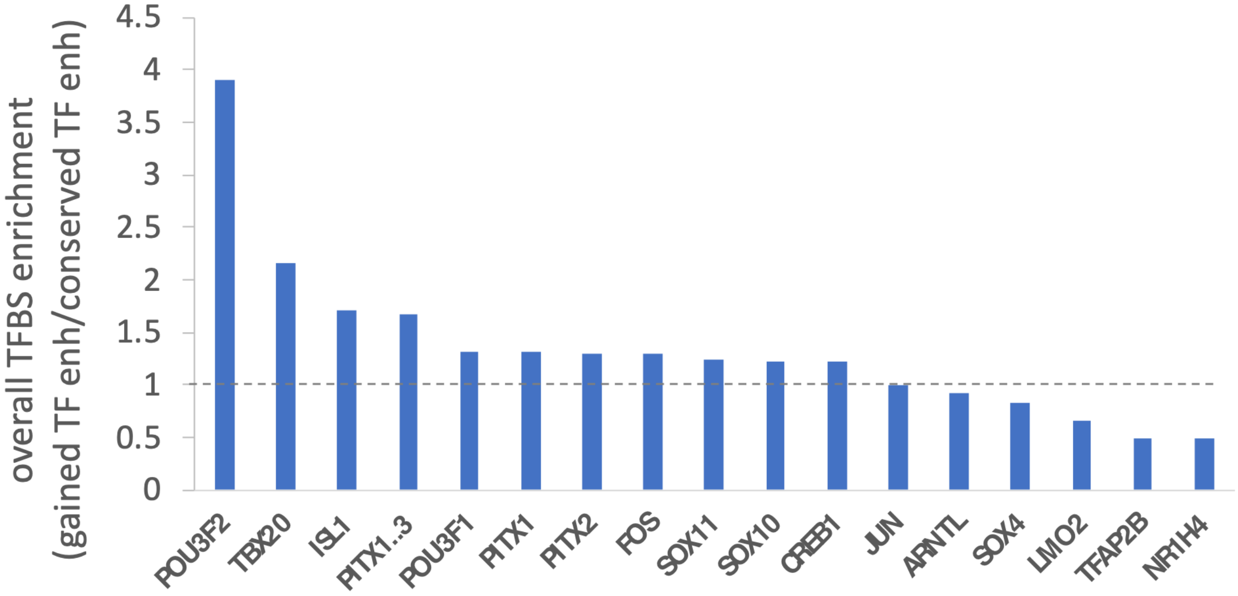

Figure S12. **TFBS enrichment of gained enhancers associated with TFs, as compared to the conserved enhancers associated with TFs.**

Figure S13

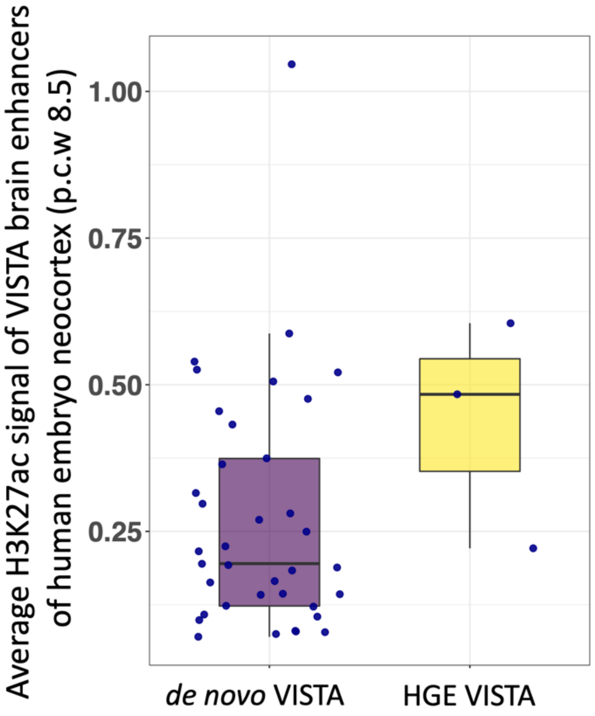

Figure S13. **Average H3K27ac signal of *de novo* VISTA brain enhancers versus that of HGE VISTA enhancers.**

Figure S14

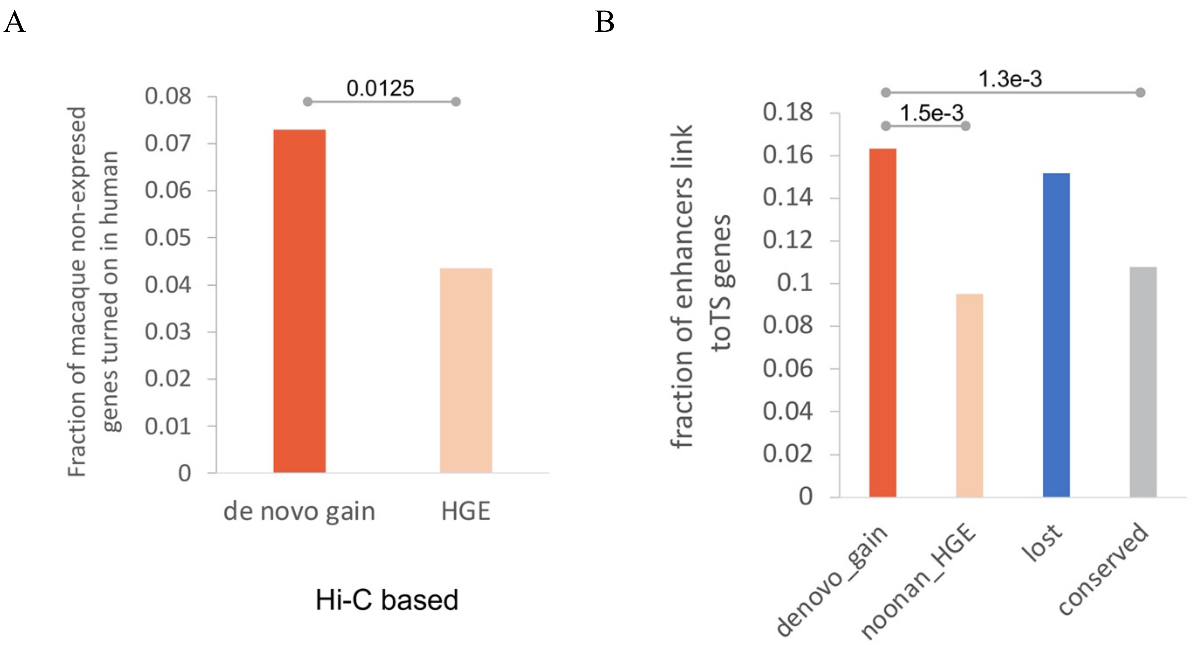

Figure S14. **De novo enhancers are more likely to turn on gene expression and regulate tissue-specific genes based on Hi-C.** (A) Fraction of enhancers in contact with genes whose RPKM < 1 in macaque and > 1 in human. The gene expression data is from the study (*26*). (B) Fraction of enhancers in 3D contact (*49*) with the most tissue-specific genes.

Figure S15

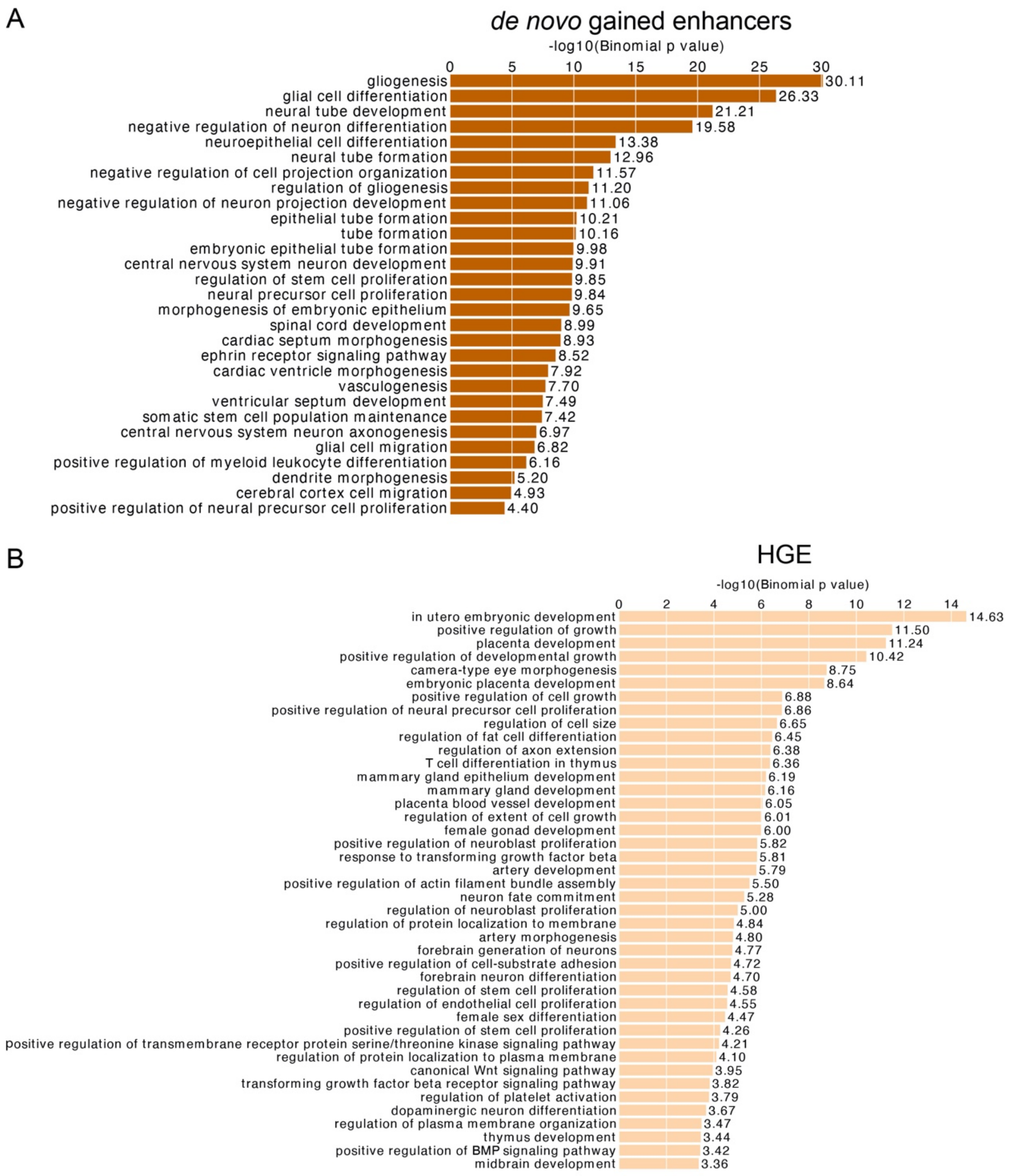

Figure S15. **Enriched GO biological processes enhancers using whole genome as the background.** (A) Enriched GO biological processes of *de novo* gained enhancers. (B) Enriched GO biological processes of HGEs.

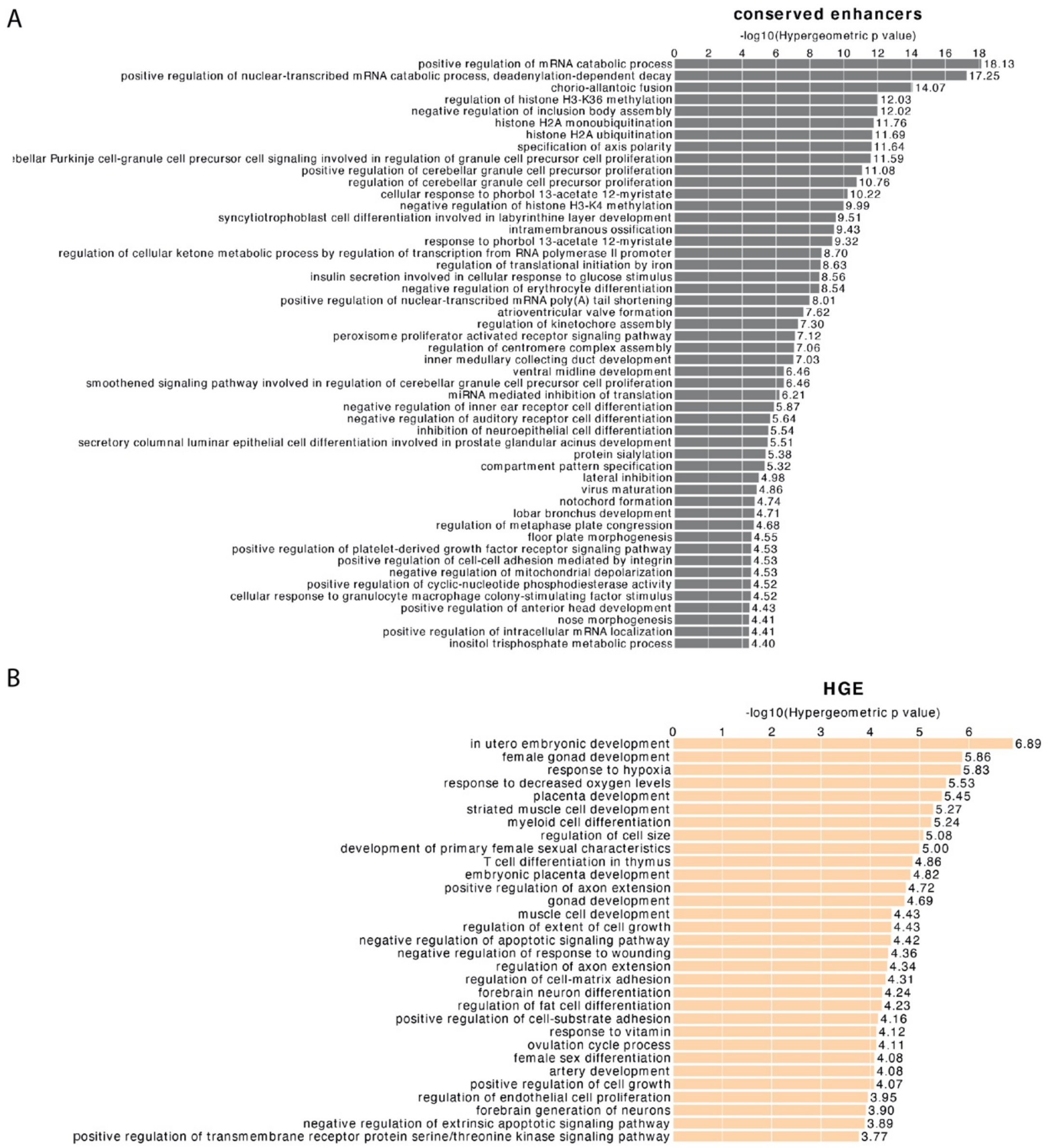

Figure S16. **Enriched biological processes of a set of enhancers, using all fetal brain enhancers (*51*) as the background.** (A) GO terms of conserved enhancers. (B) GO terms of HGEs. We apply GREAT with the single nearest gene association rule to do functional enrichment of genes near enhancers. The GO terms will be considered as enriched if it has at least 10 gene hits with FDR threshold set as 0.01.

Figure S17

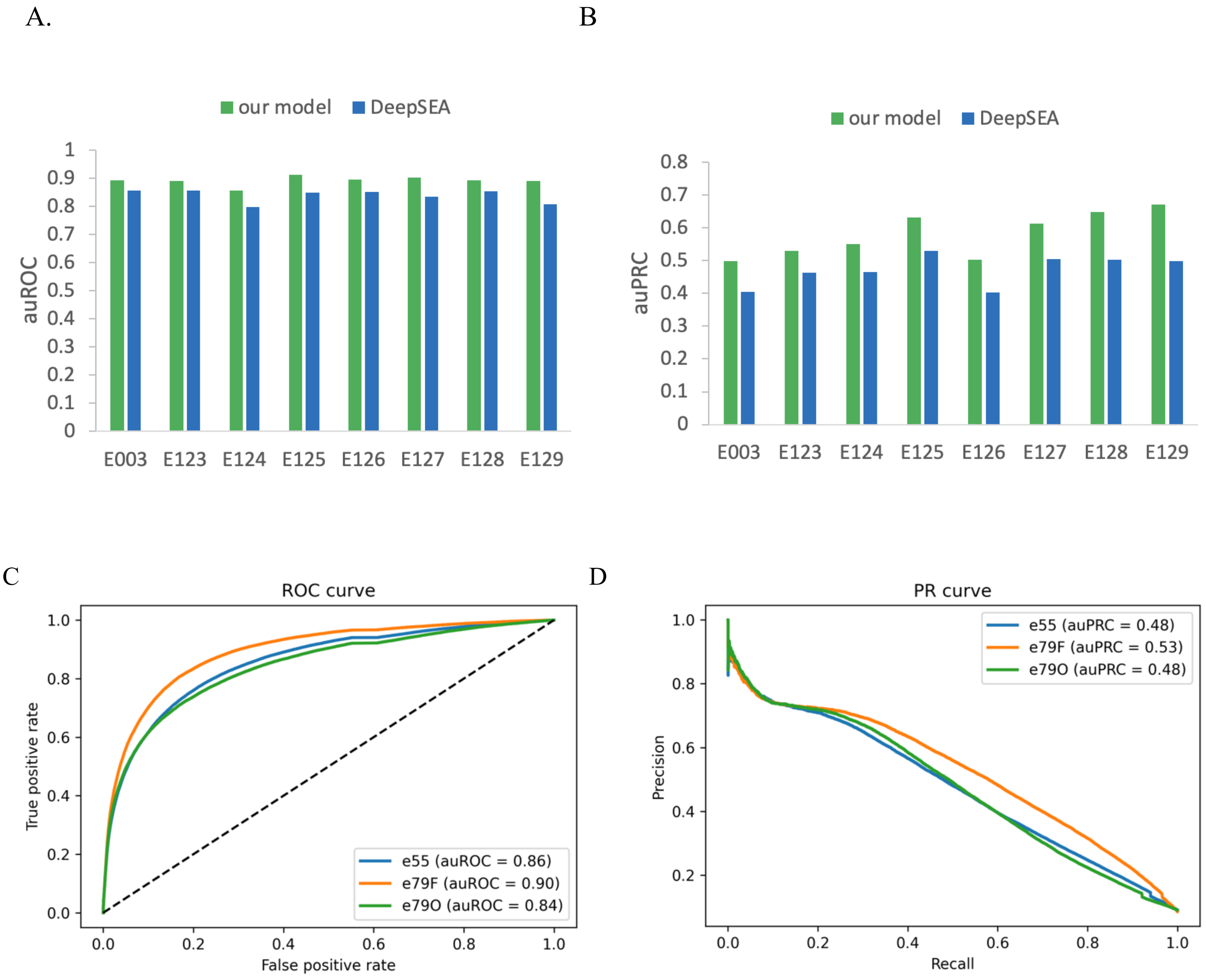

Figure S17. **Performance of the DLM.** (A) auROC and (B) auPRC of our model in predicting H3K27ac in 8 tissues which are tested by DeepSEA. (C) ROC curve of human CS23 model tested on macaque embryonic neocortex enhancers corresponding to different stages of development (e55, e79F, e79O). (D) PR curve of CS23 model tested on macaque embryonic neocortex enhancers corresponding to different stages of development (e55, e79F, e79O). The E numbers on the x-axis are the tissue IDs defined by the Roadmap Epigenomic Project. E003: H1 Cell Line, E123: K562 Leukemia Cell Line, E124: Monocytes-CD14+ RO01746 Cell Line, E125: NH-A Astrocytes Cell Line, E126: NHDF-Ad Adult Dermal Fibroblast Primary Cells, E127: NHEK-Epidermal Keratinocyte Primary Cells, E128: NHLF Lung Fibroblast Primary Cells, E129: Osteoblast Primary Cells.

Figure S18

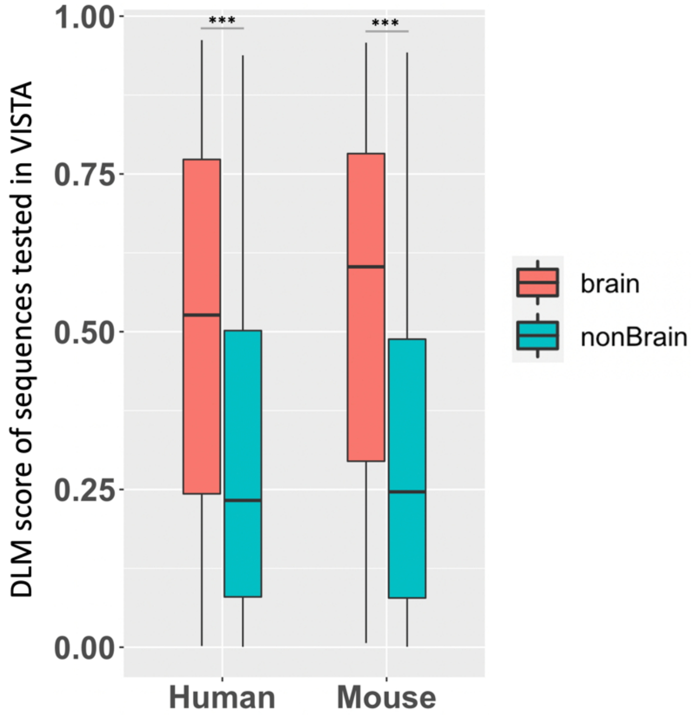

Figure S18. **DLM score of all enhancer sequences in VISTA.** *** indicates Wilcox test P-value <= 1e-5. Non-brain enhancers refer to the enhancers that were tested but were not found to be active in brain.

Figure S19

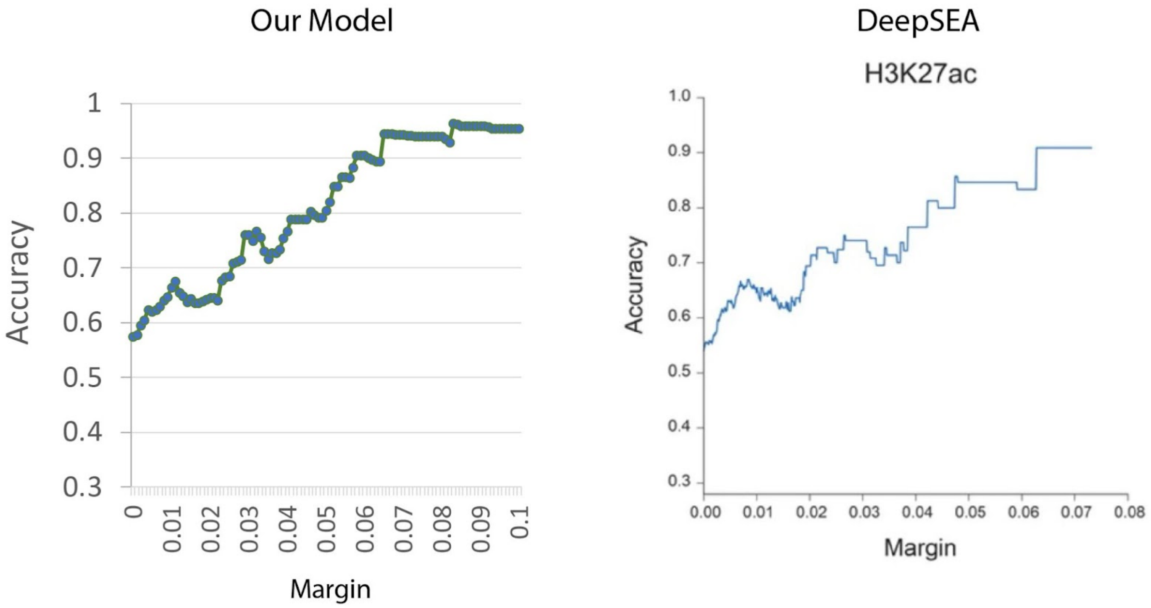

Figure S19. **Deep learning H3K27ac classifiers provided accurate prediction of allele specific effects on histone marks H3K27ac (the allele with stronger histone mark signals)**. The predictions were evaluated with histone mark QTLs identified with FDR < 0.1 in Yoruba lymphoblastoid cell lines (*72*). Margin shown on the x axis is the threshold of predicted probability differences between the two alleles for classifying high-confidence predictions. Performance is measured by accuracy (y-axis) of predicting the allele with higher read counts based on DLM score difference above certain threshold (x-axis).

Figure S20

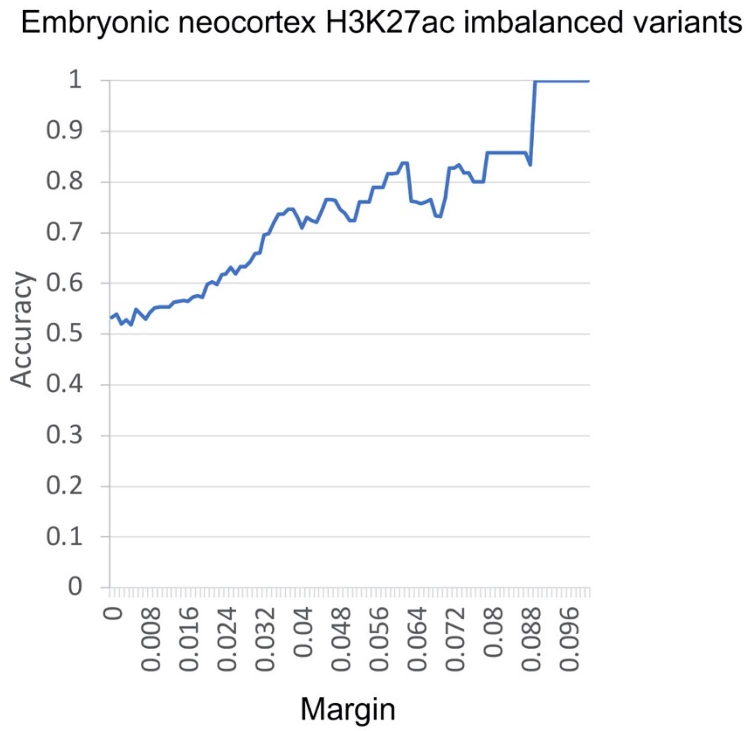

Figure S20. **The DLM of CS23 H3K27ac accurately predict allelic imbalanced heterozygous variants within CS23 H3K27ac peaks.**

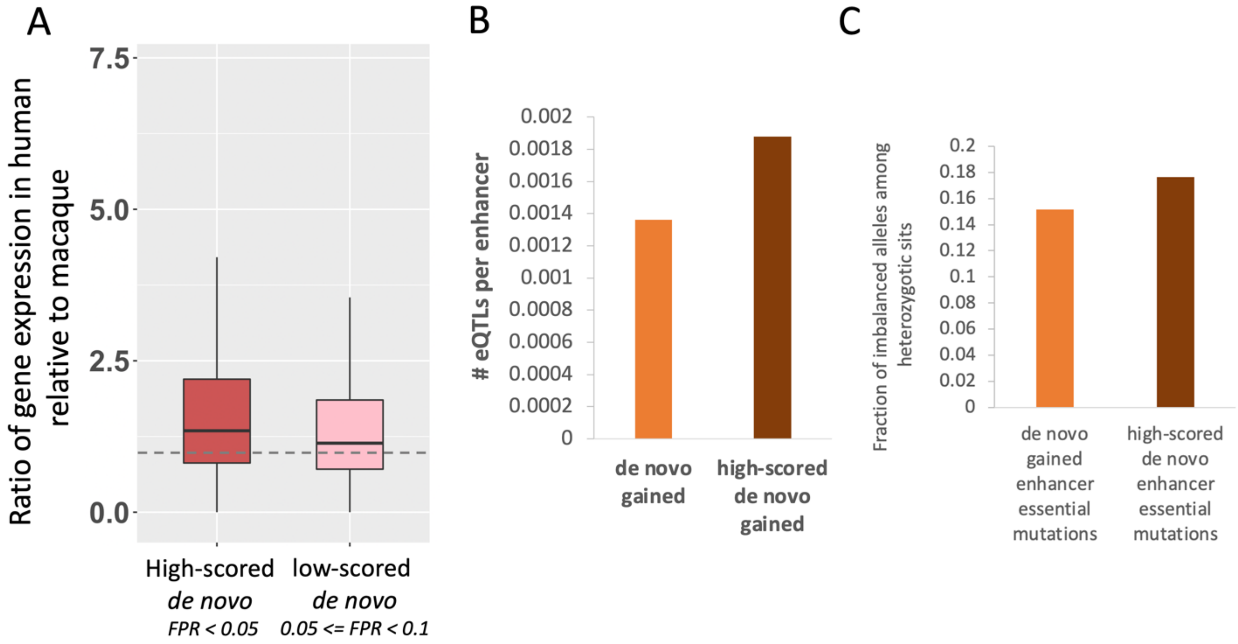

Figure S21. **A refined set of de novo gained enhancers (FPR<=0.05) exhibit stronger signal compared to the set of de novo gained enhancers (FPR <= 0.1).** (A) The expression level of genes near the *de novo* gained enhancers. (B) Average number of eQTLs per enhancer. (C) Average number of eQTLs per enhancer.

Figure S22

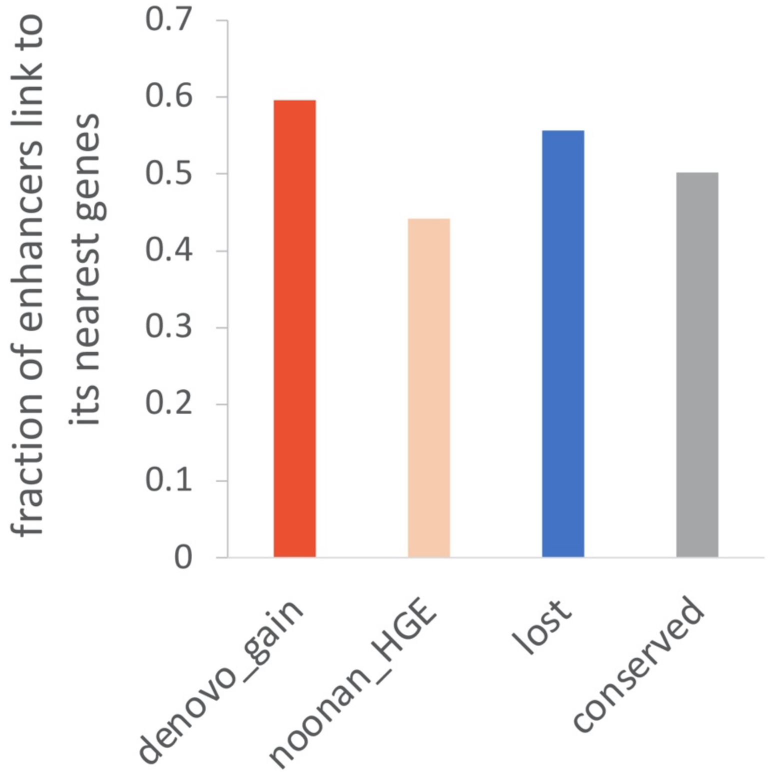

Figure S22. **Fractions of enhancers that contact their nearest gene.**

Supplementary Tables: Data S1
Supplementary Tables are provided in a single Microsoft Excel file.

Table S1. GO term enrichment of genes linked to de novo gained enhancers based on Hi-C.
Table S2. GO term enrichment of genes linked to lost enhancers based on Hi-C.
Table S3. CNS related GWAS traits overlapping conserved enhancers
Table S4. CNS related GWAS traits overlapping gained enhancers
Table S5. CNS related GWAS traits overlapping lost enhancers
Table S6. The top 2000 genes with the highest ratios of the human embryonic expression to the mean of the GTEx expression were identified as the most specifically highly expressed genes in human embryonic neocortex.
Table S7. GWAS traits overlapping essential mutations
Table S8. GWAS traits overlapping non-essential mutations
Table S9. TFBSs that are likely to be gained or lost due to essential mutations which overlap CNS-related GWAS traits.
Table S10. List of TFs whose binding sites are enriched in the de novo gained enhancers compared to the conserved ones (using both human and macaque sequences to avoid allelic bias).
Table S11. List of TFs genes near de novo gained enhancers.
Table S12. GO enrichment of genes linked to HGEs based on Hi-C.
Table S13. List of human and macaque individuals at the approximal matching developmental stages
Table S14. Architecture of DLM.
